# Combined Strengthening Techniques to Improve the Out-of-Plane Performance of Masonry Walls

**DOI:** 10.3390/ma12071171

**Published:** 2019-04-10

**Authors:** Elena Ferretti, Giovanni Pascale

**Affiliations:** Department of Civil, Environmental and Materials Engineering—DICAM, Alma Mater Studiorum Università di Bologna, 40136 Bologna, Italy; giovanni.pascale@unibo.it

**Keywords:** masonry buildings, out-of-plane strength, hammering actions, seismic retrofitting, bracing, dissipative systems, CAM system, CFRP strips

## Abstract

The purpose of this study is to improve the performance of walls under out-of-plane loads especially when subjected to the hammering action of the floors. The idea behind the paper is to provide the masonry walls with a device that behaves like a buttress, without having to build a traditional buttress. The solution presented in this paper consists of a mechanical coupling between the three-dimensional net of steel ribbons of the CAM (Active Confinement of Masonry) system and the CFRP (Carbon Fiber Reinforced Polymer) strips. Since the steel ribbons of the CAM system have a pre-tension, the mechanical coupling allows the steel ribbons to establish a semi-rigid transverse link between the CFRP strips bonded on the two opposite sides of a wall. Therefore, two vertical CFRP strips tied by the steel ribbons behave like the flanges of an I-beam and the flexural strength of the ideal I-beam counteracts the out-of-plane displacements of the wall. The experimental results showed that the combined technique inherits the strong points of both constituent techniques. In fact, the delamination load is comparable to that of the specimens reinforced with the CFRP strips and the overall behavior is as ductile as for the specimens reinforced with the CAM system. They also inspired a more performing combined technique.

## 1. Introduction

Unreinforced Masonry (URM) buildings are about 70% of the existing buildings. A large number of them dates back to periods of time when there were no building codes and the construction rules were of empirical origin. This often makes URM structures drastically vulnerable to certain combinations of loads. In particular, since the construction of these structures took place without considering the seismic actions, most of them are not able to absorb the seismic loads induced by an earthquake.

Observations from earthquakes in the past have shown that the catastrophic collapses of the URM walls that imply serious loss of life are more likely to occur in the out-of-plane direction. Since a wall undergoes an out-of-plane collapse when the earthquake direction is orthogonal to it, ensuring that the UMR walls can dissipate energy in the orthogonal direction is of paramount importance.

**Figure 1:**
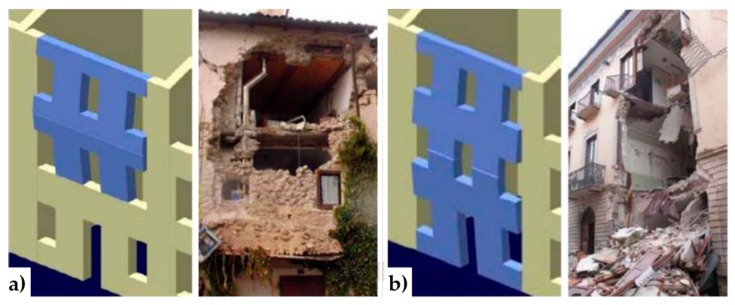
Out-of-plane collapses in the presence of an upper kerb: (**a**) partial collapse and (**b**) a global collapse.

**Figure 2:**
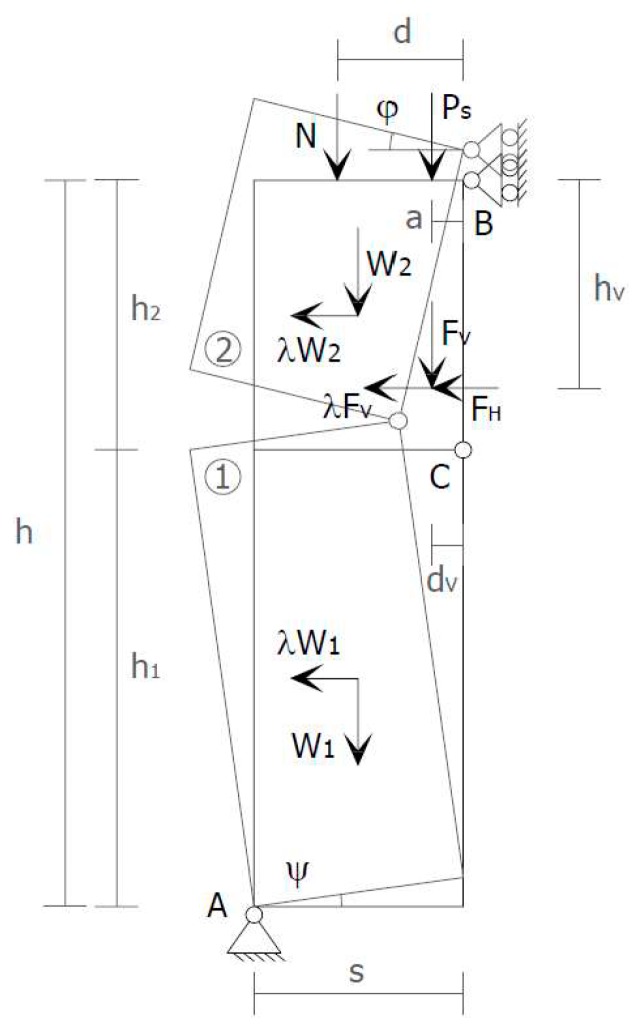
Failure mechanism for the hammering action of a floor when an upper kerb retains the wall. The internal hinge makes the system a labile scheme.

A recurrent case of out-of-plane collapse is caused by the floors of a multi-story building. Actually, due to the oscillatory nature of the earthquake forces, when the earthquake direction is orthogonal to a wall of a multi-story building, the wall receives subsequent out-of-plane thrusts from the floors, known as the hammering actions of the floors on the wall. The subsequent pulses of the earthquake may lead the wall to either overturn or break into two parts. This second failure mechanism, which is the most usual one, activates when an upper kerb retains the wall and takes place with either a partial mechanism (Figure 1a) or a global mechanism (Figure 1b). In both cases, the wall breaks with the formation of a third hinge in correspondence of one of the floors (Figure 2). As far as the meaning of symbols in Figure 2 is concerned:

- s is the thickness of the wall;
- h is the height of the wall;
- W is the self-weight of the wall;
- $P_{s}$ is the weight transmitted to the wall by the floor;
- N is the weight of upper walls and floors;
- $F_{V}$ is the vertical component of the thrusts given to the wall by arches and vaults;
- $F_{H}$ is the horizontal component of the thrusts given to the wall by floors, arches, and vaults;
- a is the distance between $P_{s}$ and the hinge in B;
- d is the distance between N and the hinge in B;
- $d_{V}$ is the distance between $F_{V}$ and the hinge in B;
- $h_{V}$ is the distance between $F_{H}$ and the hinge in B.

**Figure 3:**
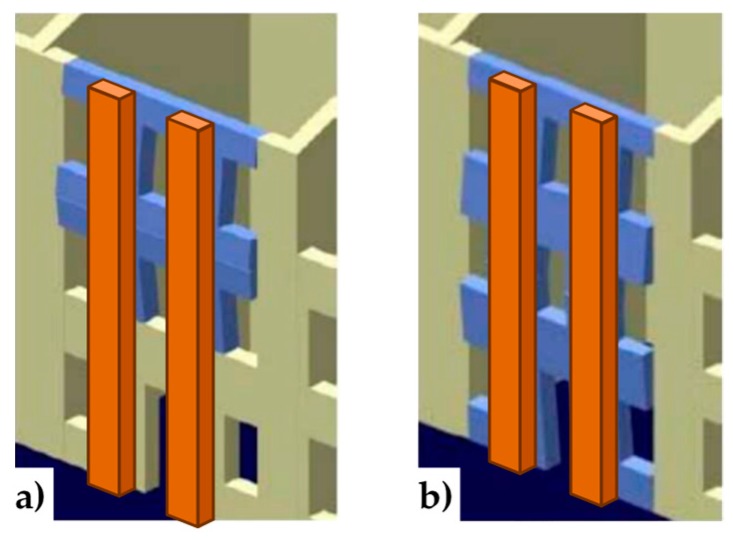
Principle of operation of buttresses to prevent collapse: (**a**) partial collapse and (**b**) global collapse.

Avoiding the mechanisms of out-of-plane collapse that originate from the hammering action of floors is one of the main concerns in modern seismic retrofitting of masonry buildings [1,2,3]. Actually, engineers have been addressing the problem of counteracting the out-of-plane displacements since the dawn of building technology. One of the first architectural means used for providing support against the lateral forces is the buttress, which is a structure built against or projecting from a wall (Figure 3). Early examples of buttresses are found on the Eanna Temple (ancient Uruk), dating to as early as the 4^th^ millennium BCE (pre-dynastic Egyptian period).

Historically, buttresses served to strengthen large walls or buildings such as churches. Nowadays, they continue to be useful in large structures such as retaining walls and dams. In these cases, and whenever buttresses counteract, or retain, the lateral force of water or the earth, they may be referred to as counterforts.

To prevent the buttress projecting too much from the wall, it is possible to make the buttress in the thickness of the wall. In this second case, it is necessary to cut the wall for its entire height, build the buttress, and restore the masonry wall around it.

Both the external and embedded buttresses are effective, but highly invasive. Moreover, the external buttresses lead to increments of mass that enhance the attraction of seismic forces and the embedded buttresses often involve significant difficulties of realization.

Over the centuries, the researchers developed many other techniques for the retrofit/strengthening of URM structures [4,5], such as base isolation, seismic dampers, surface treatments, mortar joint treatments, external steel reinforcement, post-tensioning, mesh reinforcement, reticulatus system, confinement with ring beams, tie bars, and fiber/textile-reinforced mortar. In this paper, the authors propose to recuperate the simple idea of the buttress for out-of-plane bracing of walls in masonry buildings, but using new materials and new techniques. The goal of the paper will be twofold: to build a minimally invasive embedded buttress and not to increase the final weight of the retrofitted building too much. Since one of the main concerns in retrofitting masonry buildings is to guarantee a box-type behavior, the authors have developed the new techniques as improvements of the CAM system, which is a new three-dimensional continuous retrofitting system that establishes good connections between all the structural elements of a building.

Section 2 will deal with the main features of the CAM system—some of which found a recent deepening in Reference [5]—while Section 3.2 will provide details on how the authors used the CAM system along with vertical CFRP strips to achieve a cross bracing effect in the thickness of the wall, which is comparable to that provided by an embedded buttress. In particular, the combined technique shown in Section 3.2 consists of a mechanical coupling between the CAM system and CFRP strips. Section 4 will provide some experimental evidence to show the improved out-of-plane behavior of the masonry walls retrofitted by the combined technique. Based on these early results, the authors will propose a further combined technique in Section 5. Lastly, Section 6 will deal with the conclusions.

## 2. Some Basic Notions of the CAM System

**Figure 4:**
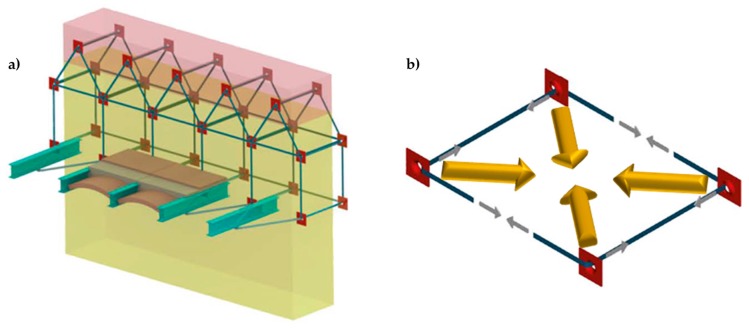
The CAM system: (**a**) the three-dimensional net of steel ribbons and (**b**) how a pre-tensioned steel ribbon compresses the masonry.

The CAM system consists of a three-dimensional net of stainless steel ribbons, which form closed loops passing through some holes, obtained by drilling the thickness of the masonry wall (Figure 4a). The use of stainless steel avoids the occurrence of corrosion problems [6]. When clamping the ribbons, a special tool provides a pre-tension to the ribbons, which then post-compress the masonry that they wrap (Figure 4b). Therefore, the CAM system is an active reinforcement technique that uses tensioned steel ribbons, which strengthen the masonry in the same way as the metallic straps strengthen the packages in heavy applications. Because of this analogy, the authors will refer to the tensioned ribbons of the CAM system as “the straps.”

Each drilled hole serves for the passage of up to six straps with different directions. Therefore, the transverse holes divide the wall into units of masonry that receive a post-compression by the pre-tensioned straps. In the case of rectangular arrangement of the holes, as in Figure 4a, the units of masonry have the shape of parallelepipeds.

**Figure 5:**
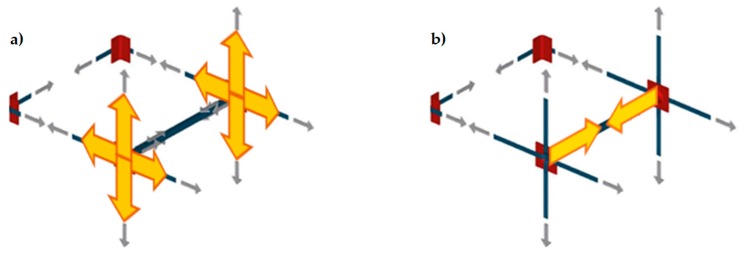
Forces acting on the nodes of the CAM system: (**a**) nodal forces in the plane of the wall and (**b**) Nodal forces along the wall thickness.

**Figure 6:**
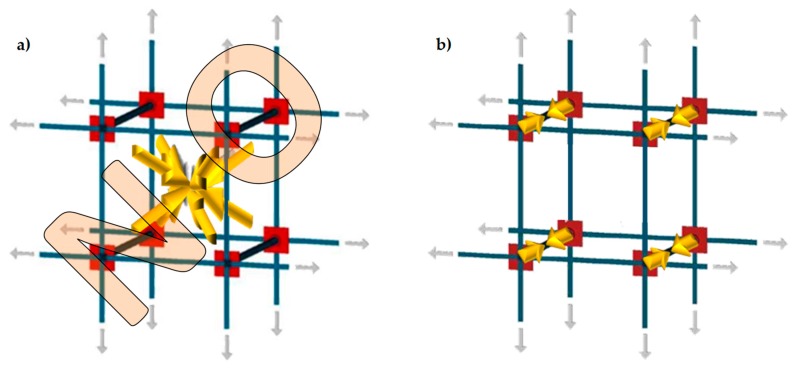
Schemes of stress transfer from the CAM system to the masonry wall: (**a**) assumption of hydrostatic state of stress and (**b**) actual scheme of stress transfer.

The discussion on the effectiveness of the CAM system provided in Reference [5] shows that the nodes of the rectangular CAM net are subjected to pairs of equal and opposite forces in the plane of the wall (Figure 5a). Consequently, they do not receive any in-plane force from the retrofitting system and do not have neither horizontal nor vertical displacements. The only nodal force not balanced by an equal and opposite force is the transverse force (Figure 5b). This means that the actual mechanism of stress-transfer from the rectangular CAM net to the masonry wall is not hydrostatic (Figure 6a), which was believed in the early studies on the CAM system [7,8,9,10,11,12,13,14]. In fact, by eliminating the balanced forces, only the transverse forces will remain, as shown in Figure 6b. Being aware of this last statement is of paramount importance for a structural engineer. In fact, one of the main consequences of replacing Figure 6a with Figure 6b is that the value of stress in the straps becomes upper bounded. In other words, there is an upper limit value of the strap stress, which it is not possible to overcome without damaging the masonry [5].

**Figure 7:**
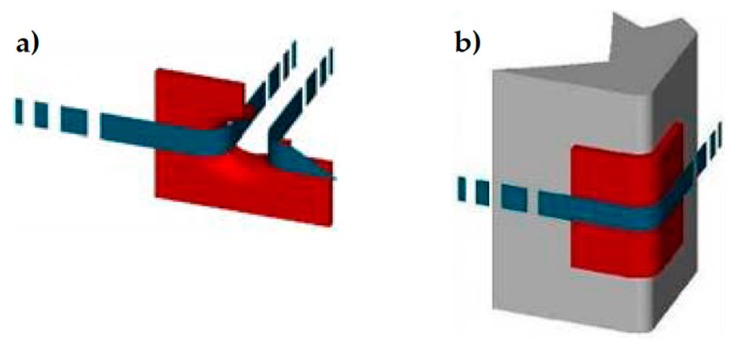
Protective elements of the CAM system: (**a**) funnel plates and (**b**) rounded angles.

The red elements in Figure 4, Figure 5 and Figure 6 are stainless steel protective elements, used in the CAM system to avoid damages at the loop corners when tensioning the straps. Their design is an integral part of the CAM patent, filed in 1999 by Dolce and Marnetto. In particular, the patent consists of funnel elements to protect the contours of the holes (Figure 7a) and rounded angles to protect the corners (Figure 7b).

**Figure 8:**
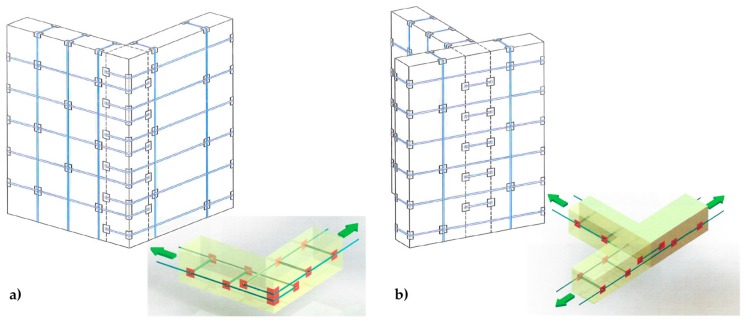
Connections between orthogonal walls, with details of the arrangement of the ribbons in the thickness: (**a**) at a building corner and (**b**) at an intersection between walls.

**Figure 9:**
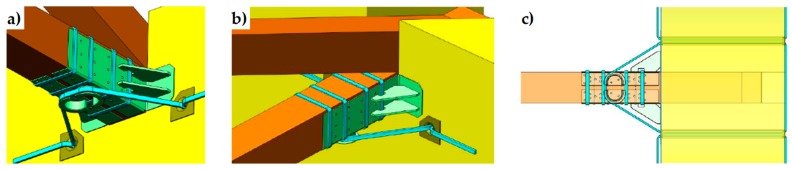
Arrangement of straps to connect perimeter walls to wooden trusses: (**a**) axonometric view from below, (**b**) axonometric view from above, and (**c**) plan view.

**Figure 10:**
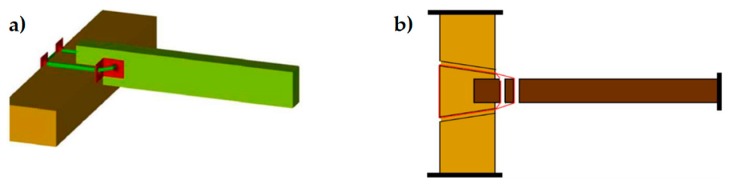
Arrangement of straps to connect perimeter walls to wooden beams: (**a**) axonometric view from above and (**b**) plan view.

In a multi-story building, the CAM system makes it possible to connect walls of different stories easily, by drilling the floors to allow the vertical loops to pass through them. Drilling is also useful at the building corners (Figure 8a) and wall intersections (Figure 8b) to connect orthogonal walls together. Moreover, it is possible to connect the perimeter walls to wooden trusses (Figure 9), wooden beams (Figure 10), and metallic beams (Figure 4a), which improves the wall-to-roof and wall-to-floor connections.

**Figure 11:**
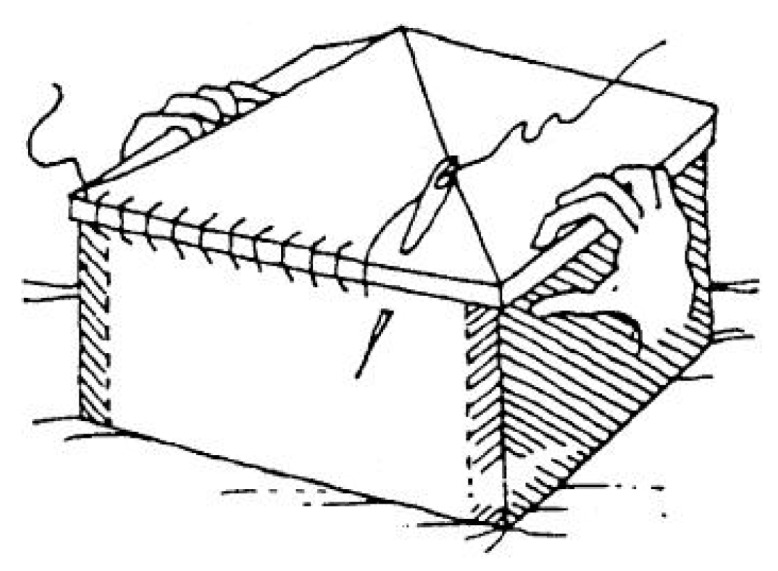
Box-type behavior supplied to the building by effective structural connections.

Improving the structural connections gives continuity to the retrofitting system, which makes it possible to connect all the structural elements together. Therefore, the CAM system is able to provide the building with an overall box-type behavior (Figure 11).

**Figure 12:**
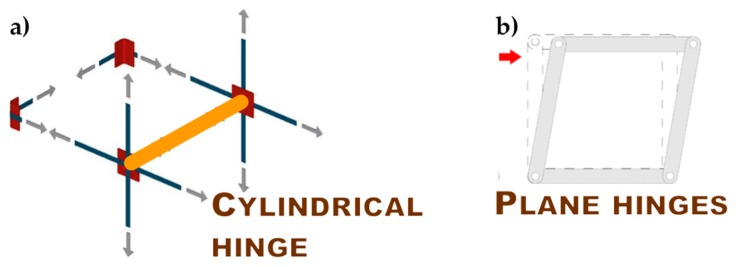
Hinged mechanisms between the ribbons of the CAM system: (**a**) in the wall thickness and (**b**) in the wall plane.

**Figure 13:**
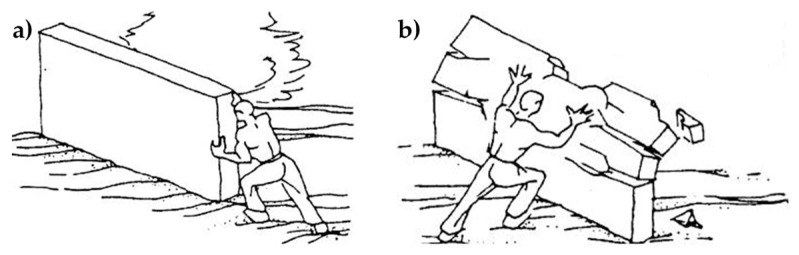
Loading of an unreinforced masonry (URM) wall: (**a**) shear loading in the midplane and (**b**) out-of-plane loading.

The holes drilled in the masonry wall behave as cylindrical hinges (Figure 12a), even when filling the holes with mortar after the retrofit. This is particularly detrimental to the rectangular arrangement of the CAM system. Actually, the straps form unbraced rectangular frame structures with hinged nodes (Figure 12b) both in the plane of the wall and the thickness of the wall. Since the unbraced rectangular frame structures are not able to withstand lateral forces and sway laterally, the rectangular arrangement of the CAM system is labile along both the in plane (Figure 13a) and transverse directions of the wall (Figure 13b). In particular, it is not able to counteract the out-of-plane loads, which is not suitable for increasing the ultimate load of collapse when the directional properties of the earthquake involve a hammering action of floors on the walls. This is why it is necessary to combine the CAM system with other reinforcement techniques to increase the strength of the wall to the hammering actions.

## 3. Techniques of Cross Bracing in the Thickness of the Wall

### 3.1. Re-Arrangement of the CAM Straps

**Figure 14:**
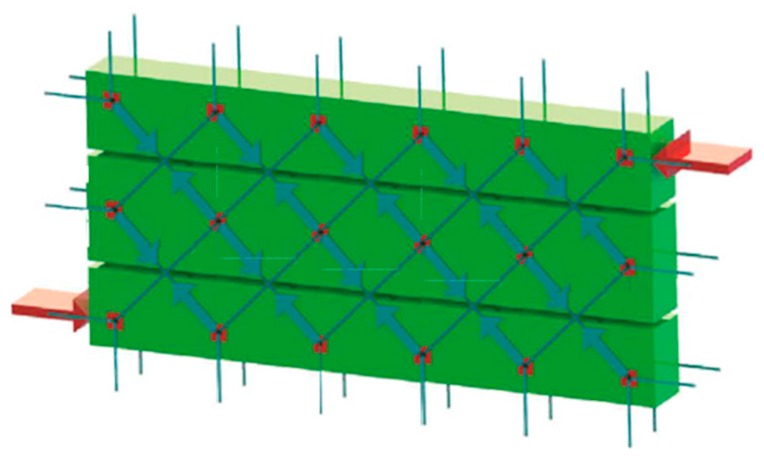
Optimized arrangement of straps to counteract the in-plane shear loads.

As shown in Reference [15], it is possible to re-arrange the CAM straps to find a statically determined strap configuration for lateral loads, in order to increase the load-bearing capacity for shear loads. In fact, arranging the straps along the two principal directions of stress for shear stress—which form angles of ±45° with respect to the horizontal direction [16]—provides a cross bracing effect in the plane of the wall (Figure 14).

**Figure 15:**
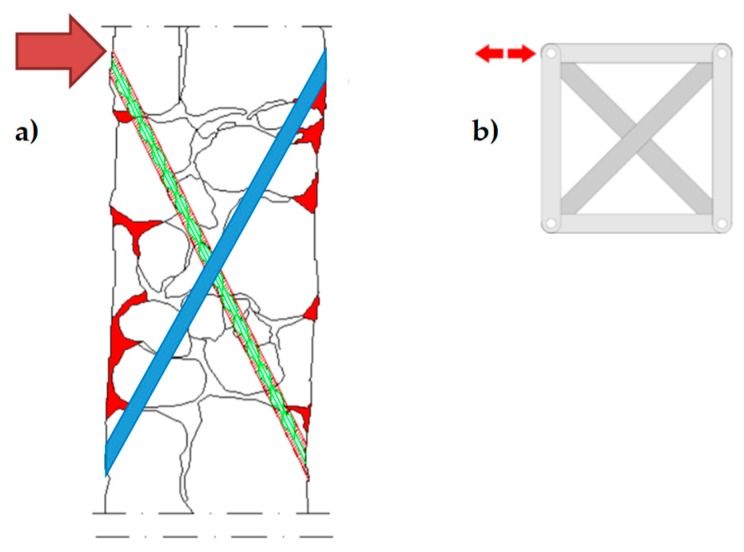
Optimized arrangement of straps to counteract the out-of-plane loads: (**a**) the ribbons of the two staggered meshes pass through the wall along inclined perforations with opposite slopes (positive slopes for the mesh drawn in blue, negative slopes for the mesh drawn in green). (**b**) The inclined ribbons act on the nodes of the CAM system as a cross-bracing act on the nodes of a rectangular frame structure with hinged nodes.

The idea of turning the straps in search of a cross bracing effect suggests a possible application to counteract out-of-plane displacements such as drilling the wall along directions not orthogonal to the wall, with positive and negative slopes alternately (Figure 15a). Though theoretically possible, this solution is impracticable because too complicated from the technological point of view. Therefore, to increase the load-bearing capacity for out-of-plane loads, it is necessary to develop some alternative solutions.

### 3.2. Application of the CAM System Together with CFRP Strips

The second technique of cross bracing in the thickness exploits the ability of the CAM system to establish a transverse link with designable stiffness. In fact, once the type of stainless steel and the cross-section of the straps have been chosen, it is possible to increase the stiffness of the transverse link by increasing the number of straps per loop (up to a maximum of four straps per loop).

**Figure 16:**
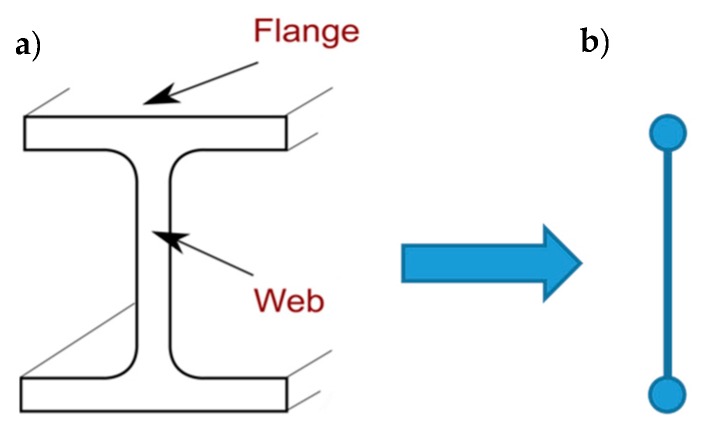
(**a**) Cross-section of an I-beam; (**b**) Ideal scheme of behavior of an I-beam in its cross-section: two point masses, linked by a stiffness constraint.

It is precisely the possibility of designing the stiffness of the transverse link that allows the use of the CAM system to build an embedded buttress. In fact, the most common way to build an embedded buttress with a traditional technique is to incorporate an FRP I-beam in the thickness of the wall, placing the two flanges vertically on the two faces of the wall to maximize the moment of inertia. As well known, the cross-section of a bent I-beam (Figure 16a) behaves as two ideal point-masses, linked by a stiffness constraint (Figure 16b). The idea behind this paper is that it is possible to reverse the path in Figure 16, moving from two masses, linked by a stiffness constraint, to an (ideal) beam under bending load. In this case, by establishing a stiffness constraint between two masses placed on the two sides of the masonry wall, it is possible to obtain an (ideal) embedded buttress without having to cut the masonry wall to insert a beam.

**Figure 17:**
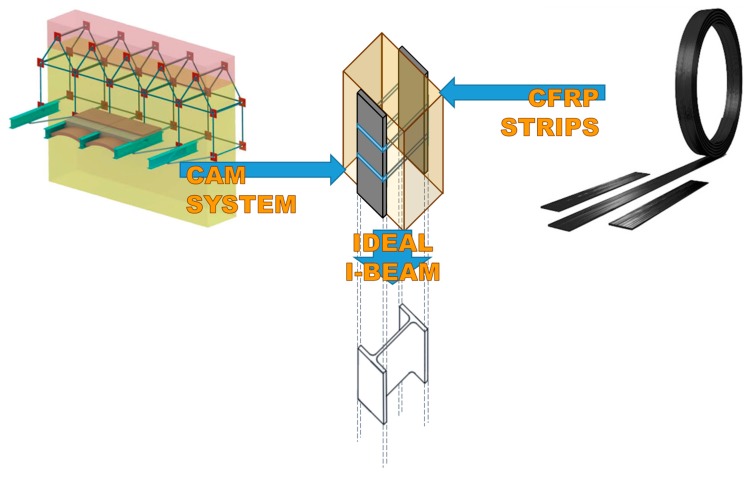
How the ribbons of the CAM system and the CFRP strips work together to provide a bracing effect in the thickness, which is similar to that given by an embedded I-beam acting as a buttress.

Following this inspiring idea, the authors decided to exploit the stiffness constraint provided by the CAM straps to link together the CFRP strips bonded on two opposite sides of a masonry wall, so that the two CFRP strips behave like the two flanges of an ideal CFRP I-beam (Figure 17). To this aim, the authors used some straps of a continuous CAM net to tie together the CFRP strips bonded on the two opposite sides of the masonry wall with one in front of the other, as shown in Figure 17. Since the CAM net crosses the floors easily, establishing effective wall-to-wall connections (Section 2), the ideal I-beam can extend to the entire height of the building, which counteracts the hammering actions of the floors.

In the explanatory scheme shown in Figure 17, the masonry wall enclosed between the two CFRP strips acts as a lost formwork, as it serves to define the distance between the two flanges of the ideal I-beam and remains within the construction. The thicker the masonry wall, the higher the web and, consequently, the greater the moment of inertia of the ideal I-beam [17].

**Figure 18:**
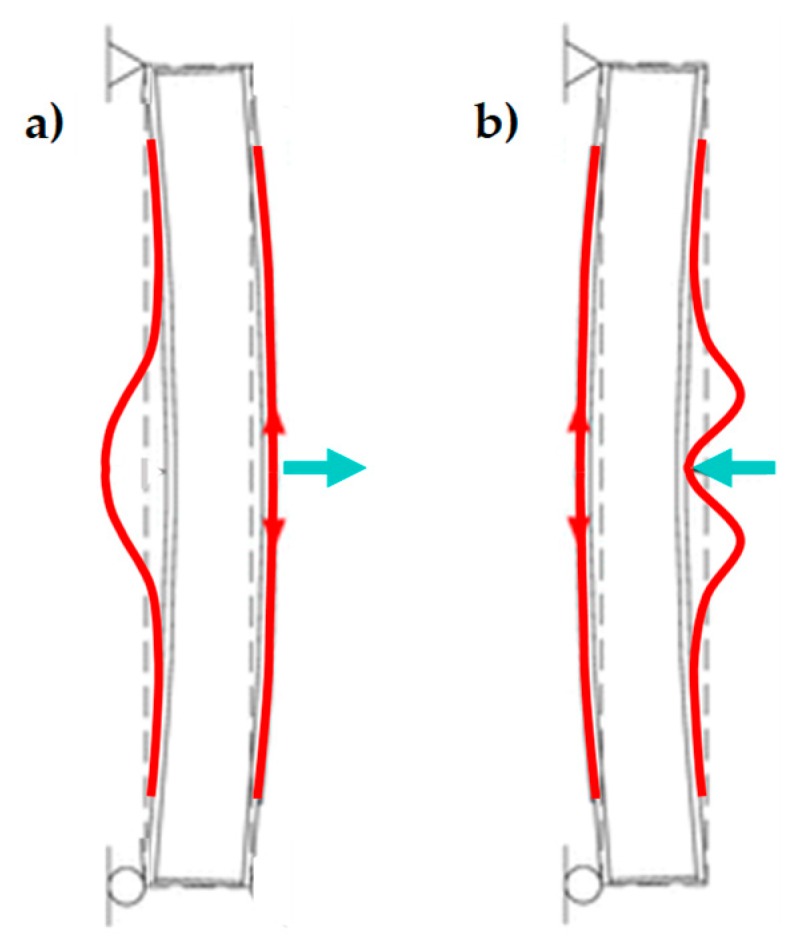
Buckling of the compressed CFRP strip for the two possible positions of the load: (**a**) load applied on the side opposite to that of buckling and (**b**) load applied on the side of buckling, pushing on the compressed CFRP strip.

Before tying the two strips of CFRP together, the strips work independently, in the sense that there is no bound between them. In particular, the ultimate flexural strength of the masonry wall depends on the strip applied on the stretched side. In fact, due to buckling, the strip on the compressed side of a bent wall undergoes delamination before the stretched strip (Figure 18).

The delamination of the stretched strip occurs when the shear forces at the strip-beam interface exceed a limit value. In absence of straps, as in Figure 18, the limit value depends on the properties of the resin, which establishes a chemical bond between the strip and the beam.

**Figure 19:**
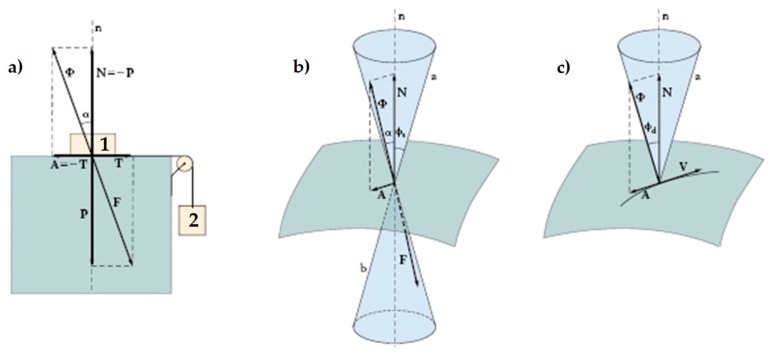
Friction forces in static and dynamic conditions: (**a**) forces at the interface between a body at rest (body 1) and its support body, (**b**) cone of static friction, and (**c**) cone of kinetic friction.

Figure 19 explains the behavior of the physical bond—which is the other possible type of bond between two bodies—when a normal force presses the two bodies together. In particular, in Figure 19:

- **P** is the weight force, exerted by body 1 on the support plane;
- **N** is the normal reaction force, equal and opposite to **P**, exerted by the support plane on body 1;
- **T** is the shear force, exerted by the hanging body (body 2) on the support plane;
- **A** is the friction force, developed by the support plane as a reaction to **T**: in static conditions, **A** is equal and opposite to **T**;
- **F** is the resultant force acting on the support plane (the component vectors of **F** are **P** and **T**);
- **Φ** is the resultant force acting on body 1 (the component vectors of **Φ** are **N** and **A**);
- $\alpha =\tan^{-1}A/N$ is the angle formed by **Φ** with the direction orthogonal to the support plane;
- $\phi_{s}$ is the angle of static friction, that is, the maximum inclination angle of the support plane before which body 1 will begin sliding on it;
- $\tan\phi_{s}$ is the coefficient of static friction, which is a dimensionless scalar value that describes the maximum ratio of the force of friction between two bodies at rest relative to each other and the force pressing them together;
- $\tan\phi_{d}$ is the coefficient of kinetic friction, which is the ratio of the force of friction between two bodies in relative motion and the force pressing them together.

Both coefficients of friction depend on the pair of surfaces in contact. For a given pair of surfaces, the coefficient of static friction is usually higher than the coefficient of the kinetic friction.

The angle $\phi_{s}$ is equal to half the aperture of the static friction cone (the right circular double cone in Figure 19b). For bodies at rest relative to each other, the angle α cannot exceed $\phi_{s}$.

If **Φ** falls within the cone of static friction, there is no relative displacement between body 1 and the support plane. When α equals $\phi_{s}$, **Φ** reaches the lateral surface of the cone of static friction. This limit condition separates the state at rest from the state of motion. As soon as **Φ** touches the lateral surface, the friction force **A** decreases so that **Φ** lies down along a generatrix of the lateral surface of the kinetic friction cone (the right circular cone in Figure 19c). Therefore, **A** and **T** are no longer vectors of equal magnitude and body 1 starts to slide along the direction of **T**.

**Figure 20:**
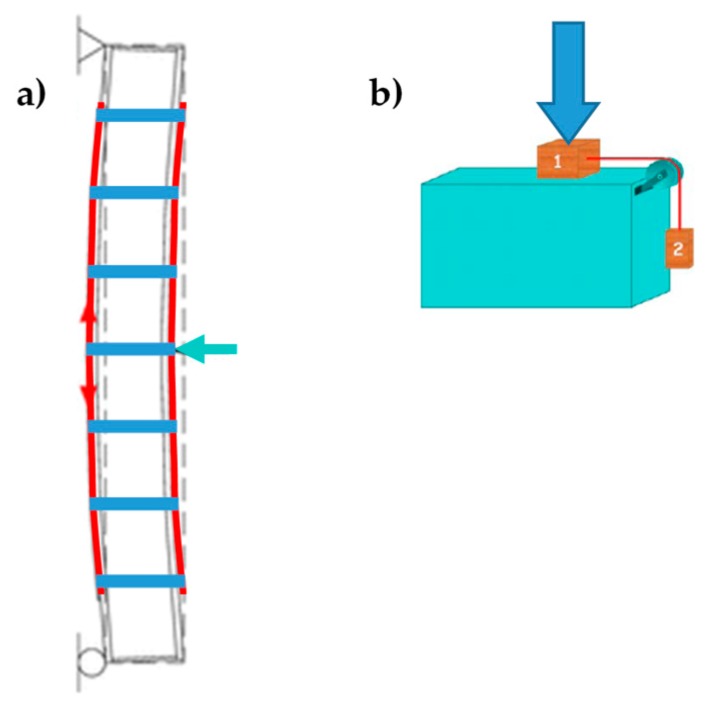
Bending of the CFRP strips tied by the CAM straps: (**a**) the straps counteract the delamination on both sides of the beam. (**b**) At the interface between a CFRP strip (body 1) and the beam (support body), the compression force (blue arrow) provided by a strap to the CFRP strip adds a physical bond that counteracts the sliding of body 1 on the body of support, caused by the sheer force (represented by the action of body 2 on body 1).

**Figure 21:**
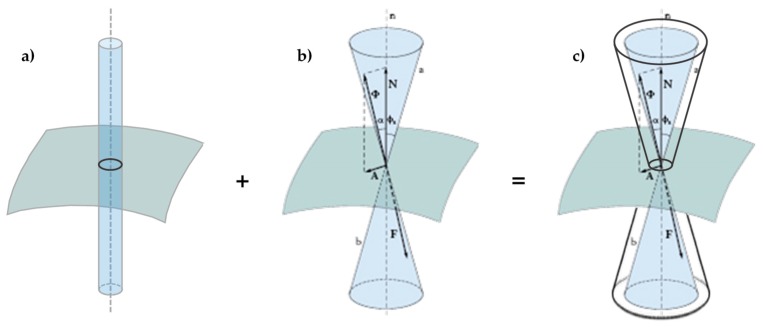
Limit surfaces in static conditions: (**a**) limit surface of the chemical bond, with the shear forces that determine the limit condition, independently of the compression forces, (**b**) cone of static friction, and (**c**) cone of cohesive static friction, which results from the combination between the limit surface of the chemical bond and the cone of static friction.

When the straps tie the CFRP strips together, the straps add a physical bond to the chemical bond provided by the resin because the pre-tension of the straps presses the CFRP strips against the masonry wall (Figure 20a) in the same way as the compression force of Figure 20b that presses body 1 against the support plane. As far as the transition from the state at rest to the state of motion is concerned, the combination between the cylinder of Figure 21a—which represents the limit surface of the chemical bond provided by the resin—and the cone of static friction (Figure 21b) results in the right circular double truncated cone of Figure 21c. Therefore, Figure 21c represents the limit surface of the cohesive physical bond, provided by the resin and the straps acting simultaneously (cone of cohesive static friction).

Compared to the chemical bond, the advantage of the cohesive physical bond is twofold:

- On the compressed side, the straps prevent the buckling of the CFRP strip, which is the main cause of delamination on that side.
- On the stretched side, the compression forces exerted by the straps on the CFRP strip modify the shape of the limit surface, from the cylinder of Figure 21a to the double truncated cone of Figure 21c. As a result, the CFRP strip can withstand higher shear forces before the head of **Φ** touches the limit surface. Since the shear forces depend on the bending load linearly, this ultimately means that the stretched CFRP strip will undergo delamination for higher values of the bending load. Therefore, the strapping delays the delamination on the stretched side.

The masonry wall benefits from the bracing effect provided by the ideal I-beam until the stretched strip undergoes delamination. After that, the actual behavior of the retrofitted system depends on the stiffness of the transverse link provided by the straps (see Section 4.5).

## 4. Experimental Program

### 4.1. Funnel Plates and Rounded Angles

**Figure 22:**
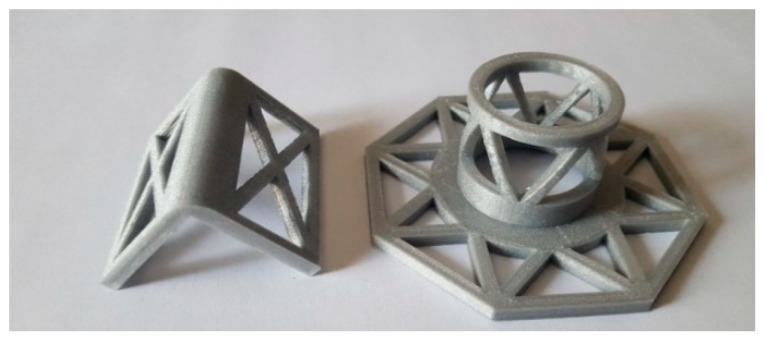
3D printed funnel plates and rounded angles.

**Figure 23:**
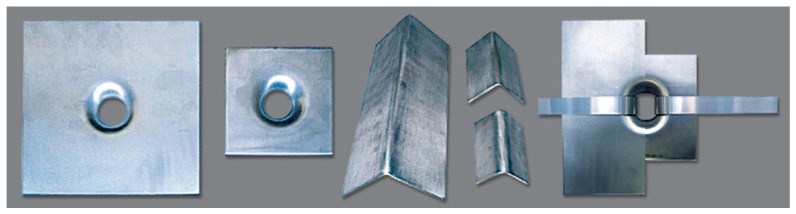
Protective elements of the CAM system [7].

The experimental program on the effectiveness of the combined technique discussed in Section 3.2 includes the design of new protective elements for the loop corners. The adopted solution, shown in Figure 22, consists of 3D printed elements, made with PLA (Polylactic Acid) filament, which substitute the CAM protective elements of Figure 23.

PLA is one of the two most commonly used filaments in FDM (Fused Deposition Modeling) 3D printing, with the other being the ABS (Acrylonitrile Butadiene Styrene) filament. The choice of the authors fell on the PLA filament for environmental reasons. In fact, the PLA filament is one of the most eco-friendly 3D printer materials available because the polymerized lactic acid comes from annually renewable resources (cornstarch, tapioca roots, sugarcane, or other sugar-containing crops). Furthermore, it requires less energy to process compared to traditional (petroleum-based) plastics.

PLA is a thermoplastic, biodegradable, and non-toxic polyester. During printing, the PLA filament emits a pleasant sugary scent and releases only carbon dioxide into the air. Moreover, one can simply discard unwanted PLA printed objects in the soil or aggressive natural environments, where they will naturally decompose. For example, an item made of PLA plastic in the ocean has a degradation time of the order of six months to two years, while conventional plastics take from 500 to 1,000 years to degrade.

It is important to point out that, although PLA will degrade in an exposed natural environment, it is very robust in any normal application or when adequately protected against degradation. Its stiffness and hardness make it similar to iron.

Both the 3D printed plates and angles have rounded external corners—where they adhere to the straps—and internal corners at 90°, to improve the coupling with the wall (Figure 22). The rounded external corner prevents the strap from bending too tightly and tearing during pre-tensioning. It is worth noting that only 3D printing can provide protective elements with these geometric characteristics since it is impossible to obtain different shapes for external and internal corners with the traditional hot forming technique used for the CAM elements in Figure 23.

**Figure 24:**
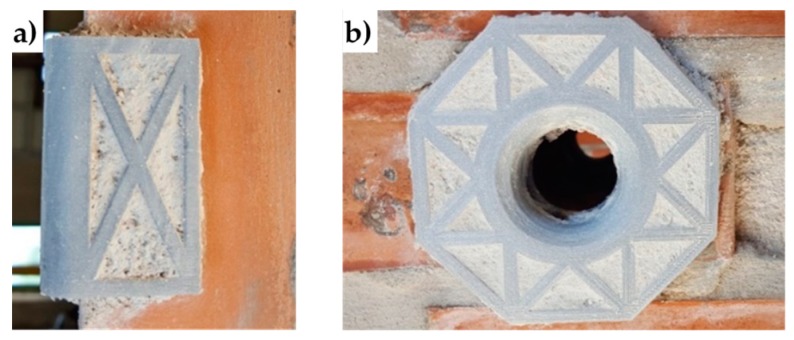
3D printed elements after placing in place: (**a**) rounded angles, and (**b**) funnel plates.

Lastly, the flat parts of the 3D printed protective elements have a truss shape. When positioning the protective elements, the mortar fills the truss structure: this improves the adherence between the masonry wall and the protective elements once the mortar has hardened (Figure 24).

### 4.2. Straps and Seals

**Figure 25:**
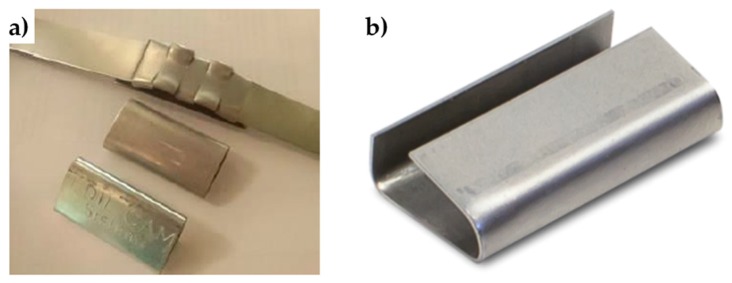
Sealing system of the patented CAM system: (**a**) steel ribbons and seals [11] and (**b**) a detail of a CAM seal.

**Figure 26:**
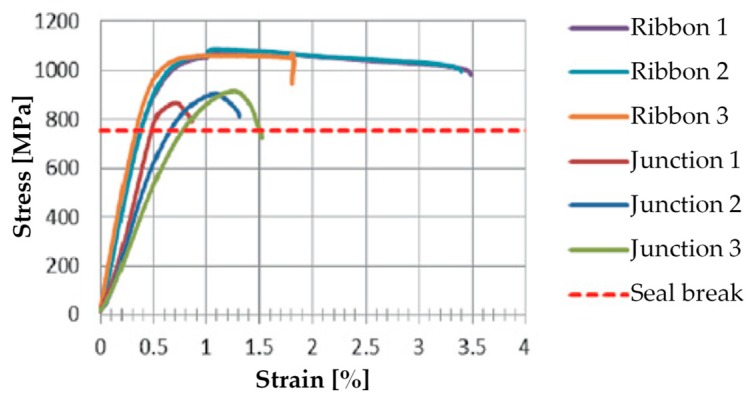
Stress/strain diagrams of the CAM ribbons, with and without seals [7].

Figure 25 shows the stainless steel straps and seals of the CAM system. The comparison between the stress/strain curves of the displacement controlled tensile tests, performed on CAM straps with and without seals (Figure 26), shows that the CAM junction is weaker than the straps. In fact, the seal of the CAM system breaks at a load that is lower than the breaking load of the strap. Consequently, the strength of a strap clamped by the CAM system is lower than the strength of the strap alone.

Figure 26 also shows that the seal modifies the stiffness of the jointed steel ribbons. In fact, the initial slope of the stress/strain curves for the jointed specimens is lower than for the specimens without junctions, which means that applying a seal decreases the stiffness of the tying system.

Lastly, the failure mechanism of the jointed specimens of Figure 26 is brittle, as the specimens break shortly after the point of maximum stress. This causes the CAM junctions to break almost suddenly.

**Figure 27:**
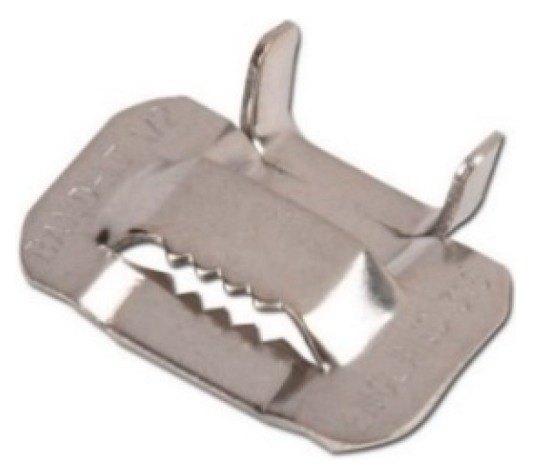
The seal used in the experimental program.

Since the brittle junctions do not allow the straps to show signs of warning against the crisis, one of the aims of the experimental program was to equip the tying system with ductile junctions. Figure 27 shows the stainless steel seal of the experimental program, used to clamp 19 mm wide and 1 mm thick stainless steel straps. Since these straps are not of the type patented with the CAM system, the authors will refer to them as “the CAM-like straps.”

**Figure 28:**
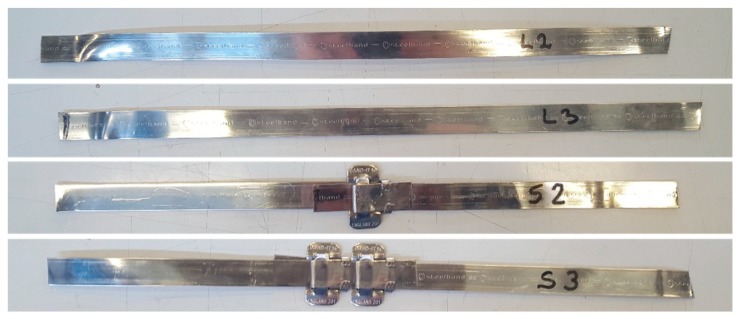
The four specimens for the characterization of the CAM-like ribbons and new junctions.

To investigate the behavior of the new junction, the authors performed tensile tests under displacement control on the following four specimens (Figure 28), where the steel ribbons were cut from the stainless steel straps of the experimental program.

**Figure 29:**
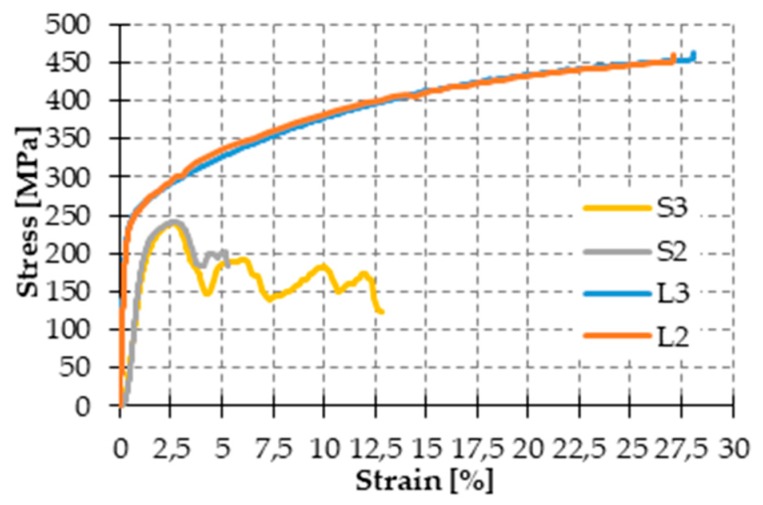
Stress/strain diagrams of the CAM-like ribbons, with and without seals.

- Specimen L2, consisting of a steel ribbon without junction;
- Specimen L3, consisting of a steel ribbon without junction;
- Specimen S2, consisting of 2 pieces of steel ribbon, fastened together by 1 seal;
- Specimen S3, consisting of 2 pieces of steel ribbon, fastened together by 2 seals;

The stress/strain diagrams in Figure 29 clearly show that the strength of the CAM-like straps is much lower than the strength of the straps used with the patented CAM system (Figure 26).

As far as the initial stiffness is concerned, the application of the seal decreases the stiffness of the tying system even for the new junction. In fact, in Figure 29, the initial slope of the stress/strain diagrams of Specimens S2 and S3 is lower than the initial slope of the stress/strain diagrams of Specimens L2 and L3.

Moreover, the yield strength of Specimens S2 and S3 is almost the same as the yield strength of Specimens L2 and L3. The maximum stress of Specimens S2 and S3, on the contrary, is lower than the maximum stress of Specimens L2 and L3. Thus, clamping by means of the seal in Figure 27 decreases the strength of the straps. Nevertheless, now the behavior of the junction is ductile, as a stage of oscillatory stress separates the maximum stress from the ultimate stress (Figure 29). Therefore, the junction no longer breaks fragilely.

**Figure 30:**
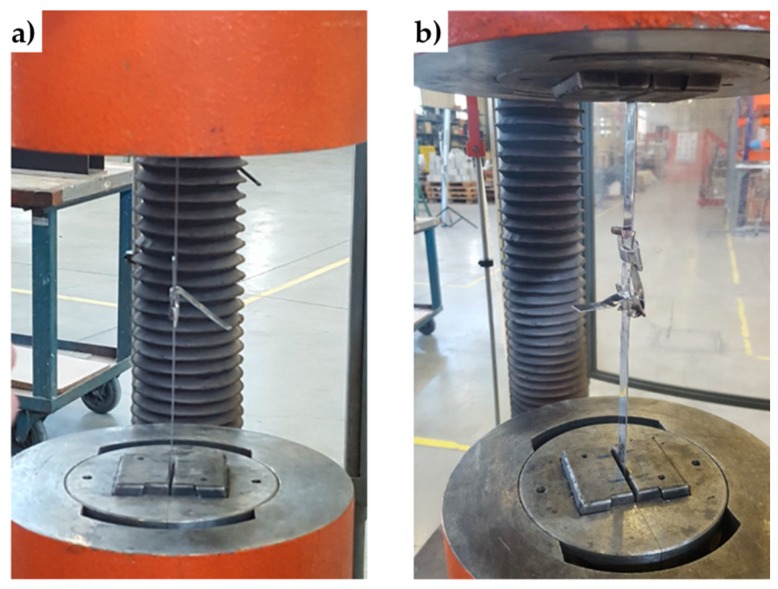
Junction unfastening: (**a**) specimen with one seal and (**b**) specimen with two seals.

The ductile behavior of the jointed steel ribbons is a consequence of the sliding that takes place—into the seal—between the ends of the straps. The sliding into the seal is also the main cause of the crisis. In fact, in the CAM system the junction fails due to the breaking of the seal. On the contrary, the new junction fails when one of the two ends slips off from the seal (Figure 30). This failure mechanism also explains why the oscillatory stage of Specimen S3 (with two seals) is longer than the oscillatory stage of Specimen S2 (with just one seal): since two seals are more effective than just one seal in counteracting the sliding, unfastening the junction of the specimen with two seals needs more time. In other words, the junction of Specimen S3 withstands a higher relative displacement than Specimen S2.

It is worth noting that, even leaving longer ends when cutting the straps, allows an unfastening delay, which happens when using two seals. In the specific case of this experimental program, however, the authors decided not to use either longer ends or two seals because an ultimate strain of more than 5% (achieved with Specimen S2) is already satisfactory for experimentation purposes.

### 4.3. Mechanical Characterization of Bricks and Mortar

**Figure 31:**
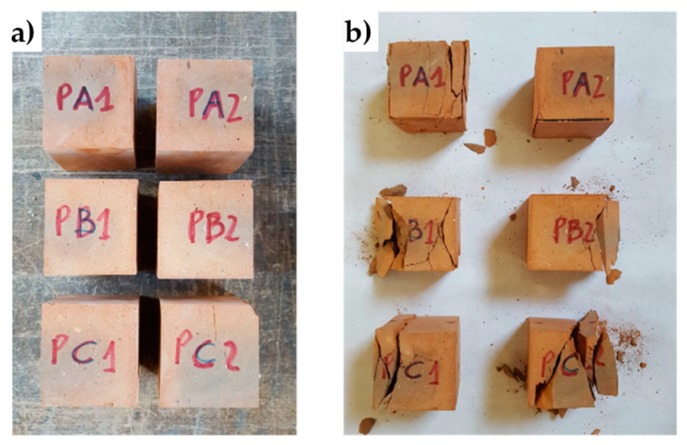
The six specimens tested for brick characterization: (**a**) before the test, (**b**) after the test.

The mechanical characterization of bricks took place by performing uniaxial compression tests on six brick specimens (Figure 31a) cut from three different bricks of the experimental program. The methods used to cut and dry the specimens and carry out the uniaxial compression tests comply with UNI EN 772-1. Table 1 collects the results of the compression tests.

The mortar of the experimental program is a single-component, fiber-reinforced, sulfate-resistant, shrinkage controlled mortar, useful for repairing and reinforcing concrete structures, mixed masonry, historic walls, and curtain walls.

**Figure 32:**
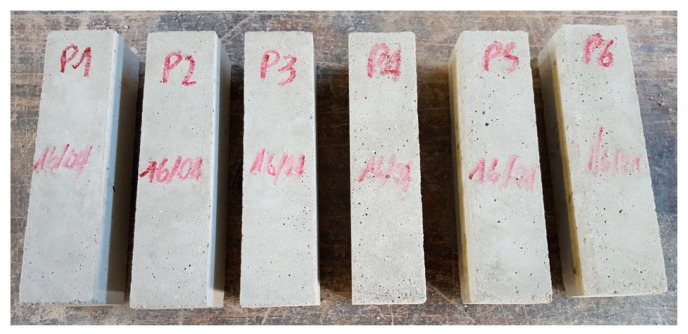
The six specimens for three-point bending flexural tests on mortar.

The mechanical characterization of the mortar complied the specifications provided by UNI EN 1015-11/2007, which establishes to classify the mortar by performing three-point bending flexural tests on prismatic specimens and uniaxial compression tests on cubic specimens. Figure 32 shows the six prismatic specimens of the experimental program. After the flexural tests, each prismatic specimen provided two cubes for the uniaxial compression test.

Table 2 shows the results of the three-point bending flexural tests on the six prismatic specimens and the uniaxial compression tests on the 12 cubic specimens (EN 196-1:2016). Since the average compressive strength is equal to 19.66 N/mm^2^, the mortar is of the M20 type.

### 4.4. Specimens W1, W2, and W3

The experimental results of this section come from the first experimental program ever performed to assess the effectiveness of the combined technique discussed in Section 3.2. To be precise, the experimental program took place in two phases: a preliminary phase of the first investigation, to check whether the mechanical coupling actually leads the two CFRP strips to behave like the two flanges of an ideal I-beam, and a second phase of improvement of the strengthening system. This section deals with the results of the first phase, while Section 4.5 will focus on the improvement of the strengthening system.

**Figure 33:**
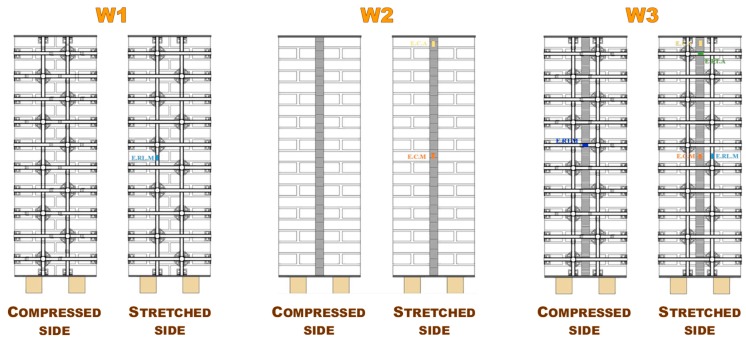
Strengthening schemes of Specimens W1, W2, and W3.

Figure 33 shows the three specimens of the phase of the first investigation (dimensions of the three brick walls: 50 × 146 × 23 cm):

- Specimen W1 is a drilled masonry wall, with the holes arranged in quincunxes. This choice minimizes the number of holes and gives rise to two three-dimensional nets of straps, staggered along the horizontal and vertical directions.
- Specimen W2 is an undrilled wall, with two CFRP strips placed side by side for each vertical centerline of the two main faces. The CFRP strips are 25 mm wide and 1.2 mm thick.
- Specimen W3 is a drilled wall, with the holes arranged in quincunxes, where some straps of both three-dimensional nets tie the side-by-side CFRP strips together, along the vertical centerlines.

**Figure 34:**
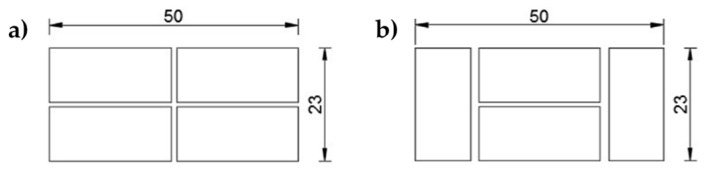
Arrangement of the bricks in the rows (all measures in *cm*): (**a**) odd rows and (**b**) even rows.

The bricks used for the experimentation are of the Bolognese type: they measure 24.5 cm in length, 5.5 cm in height, and 11 cm in depth. Figure 34 shows the arrangement of the bricks in the odd and even rows. Note that the end bricks of the odd rows are shorter, to fit the thickness of the wall. For the mechanical characteristics of the bricks, see Section 4.3.

**Figure 35:**
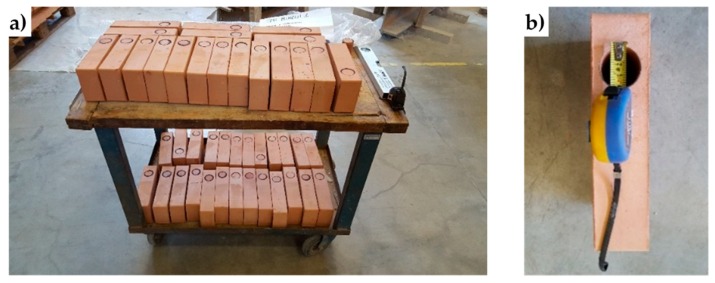
Drilling of the bricks: (**a**) preparation of the bricks and (**b**) detail of a brick after drilling.

Drilling of the bricks for Specimens W1 and W3 took place on the individual bricks (Figure 35a), before starting to build the walls. This allowed the experimenters not to face damages or stability problems of the walls during drilling. The core drill used to remove the brick cores has a diameter of 4 cm (Figure 35b).

The protective elements at the loop corners of Specimens W1 and W3 are those of the research activity discussed in Section 4.1. Moreover, the straps and the stainless steel seals used for the active confinement of Specimens W1 and W3 are the CAM-like straps and the seals described in Section 4.2.

**Figure 36:**
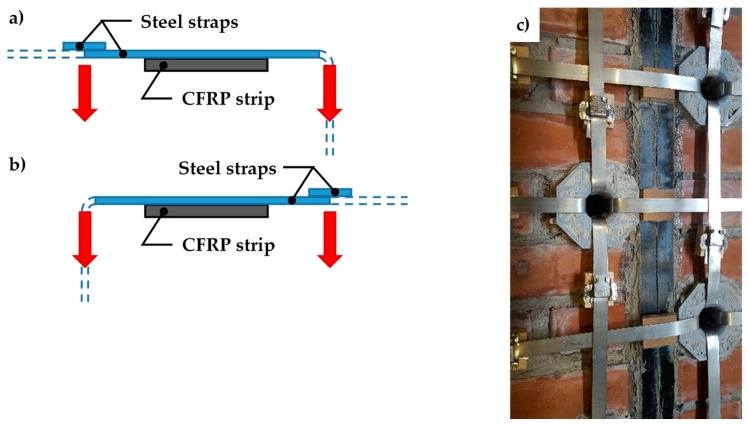
Intersections between longitudinal and transverse straps: (**a**) the longitudinal strap pushes down on the transverse strap to the left of the CFRP strip (cross-section view, not to scale). (**b**) The longitudinal strap pushes down on the transverse strap to the right of the CFRP strip (cross-section view, not to scale). (**c**) Detail of strap arrangement, with scheme sequence a, b, a.

The number of straps per loop for both specimens W1 and W3 is the minimum allowed by the CAM system: just one strap per loop. The strapping of Specimens W1 and W3 took place in two steps, first arranging all the straps spanning along the short direction (transverse straps) and, second, completing the strapping with the straps spanning along the long direction (longitudinal straps). This allows the longitudinal straps to pass over the transverse straps at the intersections between the straps (Figure 36c). As a result, the pre-tension of the longitudinal straps pushes the transverse straps against the wall, which allows the transverse straps to block the CFRP strips more firmly. Therefore, the transverse straps load the CFPR strips symmetrically, according to schemes a) and b) of Figure 36, alternatively.

**Figure 37:**
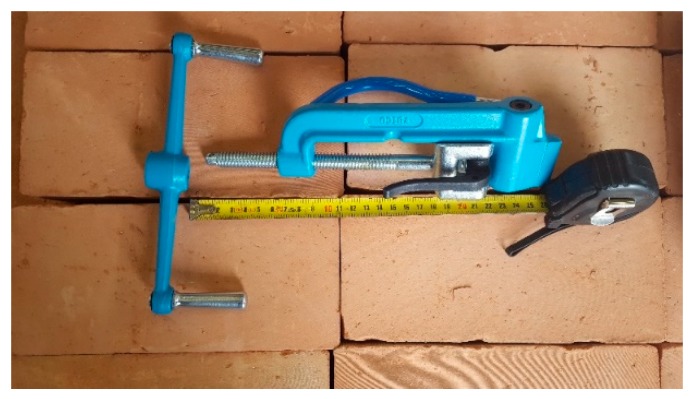
Manual strapping tool for steel.

The tool used to pre-tension the straps of Specimens W1 and W3 is the manual strapping tool for steel shown in Figure 37.

**Figure 38:**
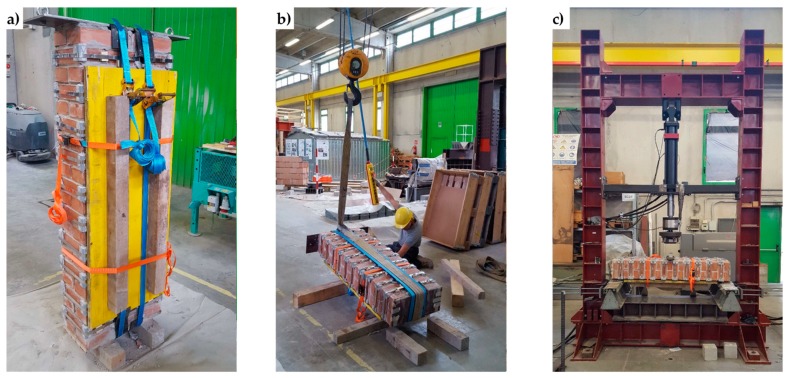
Specimen handling: (**a**) harness, (**b**) overturning, and (**c**) positioning on the testing machine.

**Figure 39:**
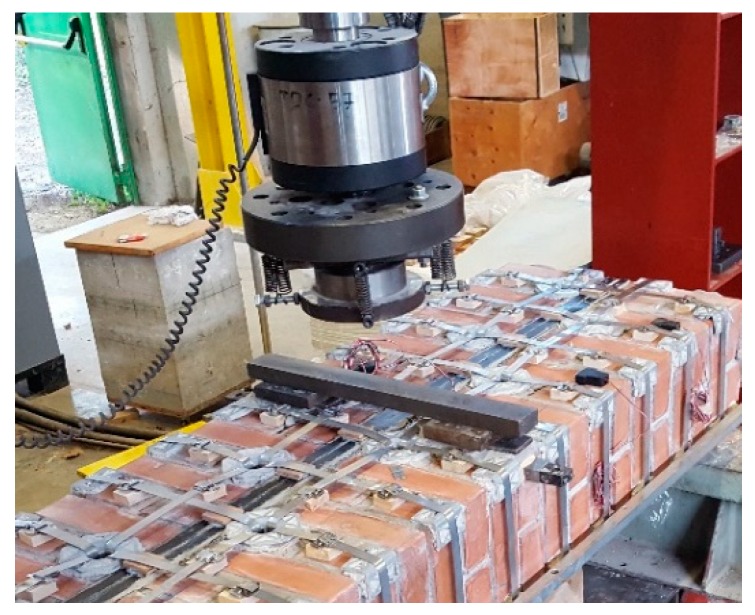
Arrangement of the flat steel bars on the central cross-section.

Since the loading piston of the testing machine applies the load vertically, it was necessary to overturn the three specimens in a horizontal configuration (Figure 38). It was also necessary to place some flat steel bars on the central cross-sections of the three specimens, in order to distribute the load given by the loading piston. The arrangement of the flat steel bars allowed the load not to compress the straps and, in Specimens W2 and W3, the upper CFRP strips (Figure 39).

The three-point bending flexural tests on the three specimens took place in displacement control, by using some Linear Variable Differential Transformers (LVDTs) to acquire the displacements at the ends and the middle point on the lower faces. Some strain gauges on both sides of the walls (Figure 33) also acquired the local deformations. The purpose of the flexural tests was to provide some initial indications on how the two CFRP strips tied by the steel straps are able to act as an embedded buttress by counteracting the vertical displacement given by the loading piston thanks to the I-beam behavior allowed by the strapping technique. In other words, the loading piston simulates the action of the floors on the orthogonal walls. The results provided by the flexural tests are only indicative because, in the spirit of a first investigation, the tests do not involve any dynamic action. Therefore, it is necessary to implement them using a dynamic analysis to draw definitive conclusions on the effectiveness of the combined technique against hammering actions.

The preliminary study [17] has already provided some early results on the effectiveness of the combined technique of Section 3.2 in counteracting the hammering action of the floors on orthogonal walls. In this case, the authors will focus on the post-delamination behavior of a masonry wall after retrofitting by the combined technique to answer the question on whether the stiffness of the transverse link modifies the post-delamination behavior or not.

The increase in ductility is the most important result with regard to the out-of-plane behavior of strapped masonry walls. Actually, since the strength of the steel ribbons is much greater than the masonry strength, the straps continue to wrap masonry even after masonry crushing. This is of fundamental importance in real buildings since people do not risk that some part of the structure will hit them, due to the building collapse. Therefore, the strapping acts as a reinforcement system before the structural damage occurs and a protection device after the structural damage had occurred.

**Figure 40:**
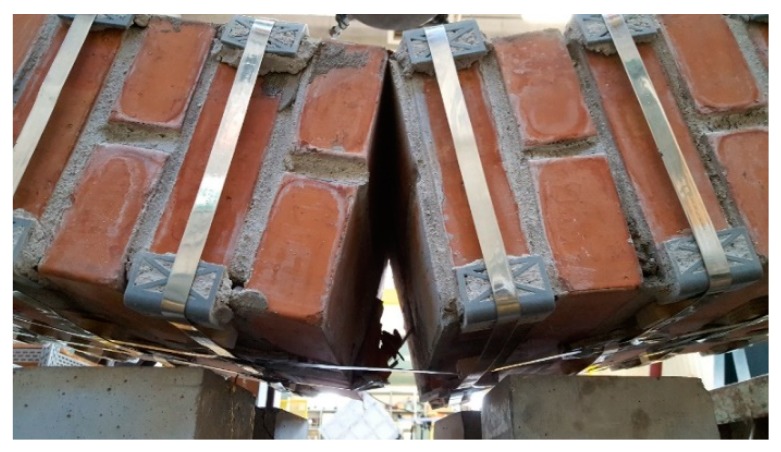
The internal hinge of Specimen W1, which has formed close to the loading piston.

**Figure 41:**
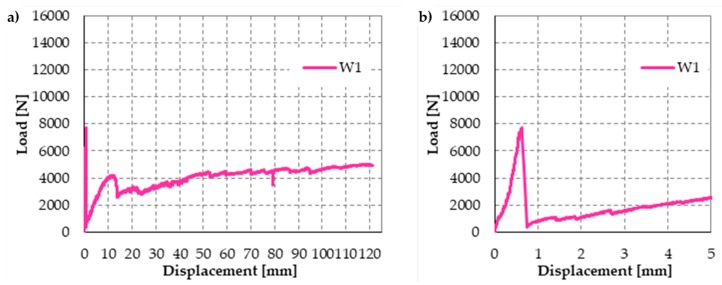
Load/displacement diagram of Specimen W1: (**a**) complete diagram and (**b**) detail of the first peak of load, with a magnified scale factor for the displacement axis.

The mechanism of safeguarding life allowed by strapping is particularly evident in Figure 40, which is a snapshot of Specimen W1, taken at the end of the test. Even if the internal hinge of Figure 2 has formed and provided a large relative rotation, the longitudinal straps still keep the beam in equilibrium. Actually, Specimen W1 never collapsed during the flexural test. To avoid instrumentation damages, the operator had to stop the test for a vertical displacement of the loading piston equal to 121.063 mm (Figure 41a), while the specimen would have withstood further increases in vertical displacement. When the operator stopped the flexural test, the specimen was still resisting a load of 5 kN, which is about 65% of the hinge formation load (7.7 kN) and the load was still increasing.

Due to the low pre-tension stress supplied to the straps, the load of hinge formation is the same for the strapped and non-strapped masonry walls. Therefore, the first-peak load of 7.7 kN is the failure load for plain specimens.

**Figure 42:**
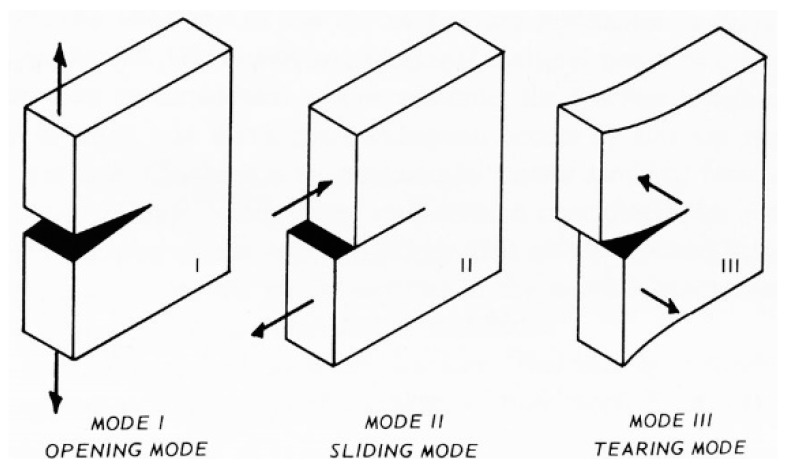
The three modes of failure in fracture mechanics.

Furthermore, the final vertical displacement (121.063 mm, see Figure 41a) is about 192 times the vertical displacement at the first peak (0.630 mm, see Figure 41b), which is the vertical displacement at failure for plain specimens. This makes the opening of the internal hinge ductile. Lastly, the vertical displacement immediately after the first peak in Figure 41a,b (0.751 mm) is almost the same as the vertical displacement at the first peak. In other words, the rotation around the internal hinge at the formation of the hinge itself was almost nil. After that, the crack under the internal hinge opened very slowly in Mode I (opening mode, see Figure 42) in a controlled manner [18]. No straps broke during crack opening. Therefore, the tying system is fully adequate to safeguard life even if the strength of the CAM-like straps is much lower than the strength of the patented CAM ribbons (see Figure 26 and Figure 29).

**Figure 43:**
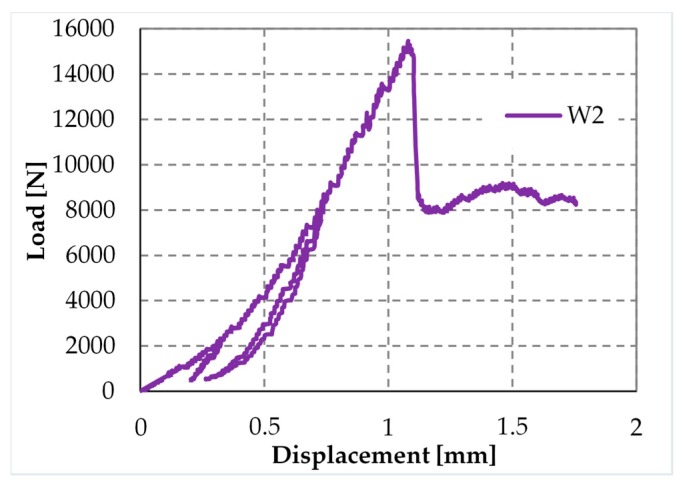
Load/displacement diagram of Specimen W2.

Figure 43 shows the load/displacement diagram for Specimen W2, with a maximum load that is twice the maximum load of Specimen W1. When compared with the load/displacement diagram in Figure 41a, Figure 43 shows that the CFRP reinforcement makes the masonry wall stronger, but also much more brittle than the steel straps.

**Figure 44:**
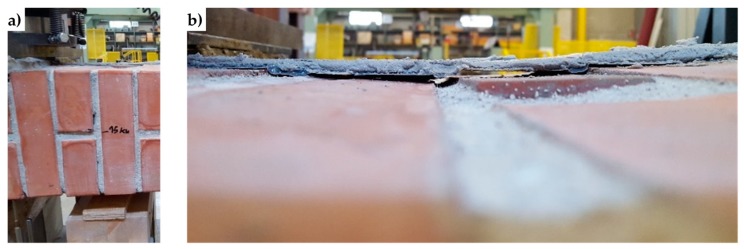
Delamination of the compressed CFRP strip: (**a**) length of the crack for the delamination load on the compressed side (15 kN) and (**b**) detail of delamination, just above the crack.

**Figure 45:**
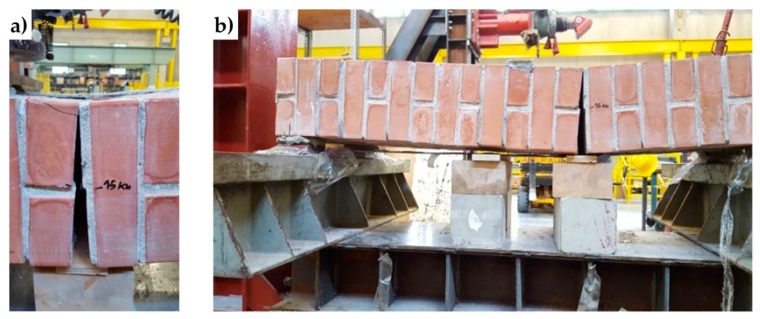
Delamination of the stretched CFRP strip: (**a**) opening of the crack (Mode I) for the delamination load on the stretched side and (**b**) detail of delamination on the stretched side.

The peak in Figure 43 corresponds to the delamination of the compressed strip, which detached itself starting from the localization cross-section of the internal hinge. Figure 44 is a snapshot of the cross-section where the internal hinge localized, taken at the maximum load (peak load of Figure 43). In particular, the mark in Figure 44a shows the crack length for a load of 15 kN, while Figure 44b is a detail of the CFRP strip delamination, initiated on the compressed side, above the crack of Figure 44a. As shown by the mark in Figure 44a, initially, the crack involved only half the thickness of the specimen. Then, the cross-section underwent a short phase of localized deformations that leads to the increase in vertical displacement—at an almost constant load—which characterizes the diagram after the peak of Figure 43. This is the phase in which the cross-section behaved as a plastic hinge. The plastic phase ended with the delamination of the lower CFRP strip, which detached itself starting from the end that was farthest away from the internal hinge (Figure 45b).

**Figure 46:**
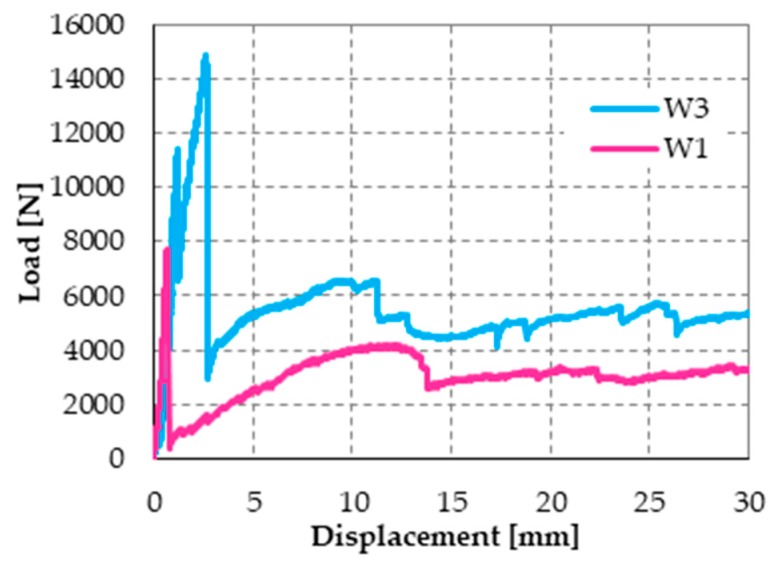
Load/displacement diagrams of Specimens W1 and W3: displacement field truncated at the value of 30 mm.

The effect provided by the two reinforcement systems that act simultaneously on the masonry wall is twofold since the combined technique increases both the strength and the ductility. In fact, the maximum load for Specimen W3 (Figure 46) is comparable to that given by the CFRP strips alone (about twice the failure load of the masonry wall) and the post-peak behavior of Specimen W3 is as ductile as that offered by the steel straps alone.

It is worth noting that, while the increased ductility of the masonry wall leads to high displacements, it does not involve a high displacement rate, which could be dangerous for people standing in a real building. In fact, when the internal hinge forms at the maximum load of Specimen W3, the beam finds a new equilibrium configuration without causing a significant increase in the vertical displacement, since the pre-peak displacement is almost the same as the post-peak displacement (Figure 46). This means that the relative rotation between the two hinged cross-sections is initially negligible. Moreover, the relative rotation increases in a controlled manner for further displacements of the loading piston, as the steel straps provide the hinge with a plastic behavior. Therefore, the straps allow the internal hinge to achieve high relative rotations without ever losing equilibrium. In other words, Specimen W3 moved along a path of stable equilibrium throughout the duration of the flexural test.

The post-delamination behavior of Specimen W3 (diagram in Figure 46 after the maximum load) shows that the steel straps retain the delaminated strip, which allows the wall to withstand loads higher than the post-peak loads of Specimen W1 (retrofitted only with steel straps). This means that the wall can benefit from the strengthening effects of both CFRP strips even after the delamination of the stretched strip, although only in part. In other words, the I-beam behavior of the combined technique does not end with the strip delamination. It survives the delamination with a decreased stiffness, which depends on the stiffness of the transverse link. Therefore, increasing the number of straps per loop should increase the load-bearing capacity after delamination since it increases the stiffness of the transverse link.

To verify this latest statement, the authors restored Specimen W3 (after testing), increased the number of straps per loop, and tested the restored specimen by performing a further three-point bending flexural test in the displacement control. This also allowed the experimenters to evaluate the effectiveness of the combined technique to restore a damaged structural element.

The new label of Specimen W3 after restoration and strapping is “Specimen W4.”

### 4.5. Specimen W4

As anticipated in Section 4.4, the main target of the second phase of the experimentation was to increase the load-bearing capacity after delamination, by increasing the number of straps per loop. This made it necessary to define a new strapping criterion since the improvement of the strapping system can occur in two different ways: by adding the same number of straps to each loop or by diversifying the number of straps per loop. The authors opted for the second possibility because, since the load-bearing capacity after delamination depends on the stiffness of the transverse link, provided by the transverse straps, it seems reasonable to increase only the number of transverse straps.

The diversified improvement is the most useful solution to solve a further problem: the excessive load drop after delamination of the CFRP strips (see the load/displacement diagram of Specimen W3 in Figure 46). In fact, excessive load drop may cause several instability-related issues and load redistribution in the building, which may trigger the collapse of adjacent structural elements. It is worth noting that, due to the box-type behavior provided to the building continuously, the three-dimensional net of straps protects the building from global collapse in any case. Nevertheless, it is always preferable to avoid excessive overloads and localized damages to adjacent structural elements, whenever possible.

**Figure 47:**
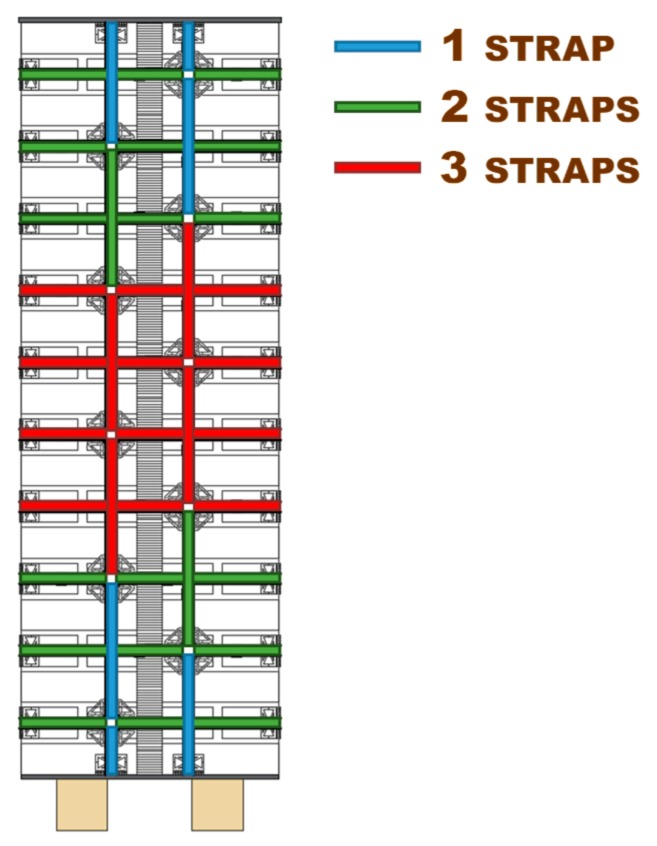
Scheme of strapping for Specimen W4.

This second time, the diversified improvement involved both the longitudinal and transverse straps. The adopted solution to limit the excessive load drop after delamination consists in using a greater number of longitudinal straps where the bending moment of pre-delamination is greater—that is, close to the middle cross-section (Figure 47)—since the probability of formation of the internal hinge is higher where the bending moment is greater. With this choice, the greater number of longitudinal straps will increase the load-bearing capacity of the beam at the formation of the internal hinge, which decreases the load drop. Moreover, since the load of post-delamination belongs to the post-delamination path, the increased number of transverse straps cooperates to increase the residual load after delamination, which further decreases the load drop.

The preparation of Specimen W4 took place as follows:

- Removal of all damaged straps of Specimen W3;
- Cleaning of the specimen surface, in correspondence of the delaminated CFRP strips;
- Removal of the mortar on the cross-section where Specimen W3 opened into two parts;
- Restoration of the specimen integrity, by walling together the two parts of Specimen W3;
- Curing of the mortar on the restored cross-section;
- Bonding of new CFRP strips on both sides of the specimen;
- Strapping of the specimen, according to the scheme of Figure 47, by positioning the longitudinal straps over the transverse straps (like for Specimens W1 and W3).

As discussed earlier, the improved strapping scheme in Figure 47 has the dual task of increasing the load-bearing capacity after delamination and decreasing the load drop at delamination. In particular, the greater number of transverse straps will increase the delamination load and improve the load-bearing capacity after delamination (starting from the load of post-delamination), while the greater number of longitudinal straps will decrease the load drop at delamination. The number of transverse straps is greater where the number of longitudinal straps available to block them is greater, which occurs close to the middle cross-section (Figure 47). After restoration and strapping, the experimenters inverted Specimen W4 to load the face that was on the stretched side of Specimen W3.

**Figure 48:**
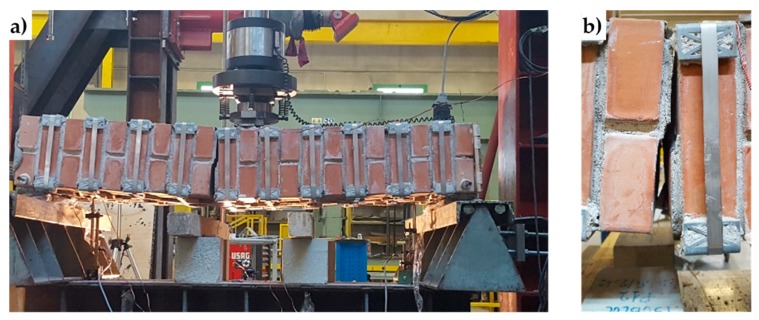
Specimen W3 at the end of the flexural test: (**a**) overview and (**b**) the failure cross-section, where the crack propagation occurred in both Mode I (opening mode) and Mode II (sliding mode).

**Figure 49:**
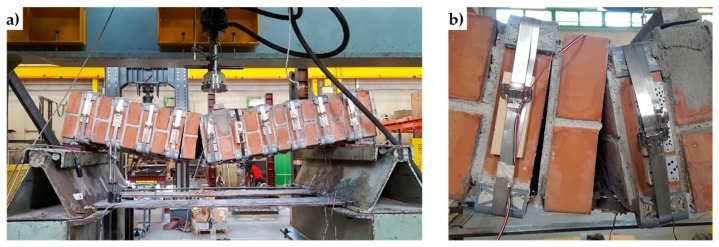
Specimen W4 at the end of the flexural test: (**a**) overview and (**b**) the two failure cross-sections, with crack propagation in Mode I on the left failure cross-section and crack propagation in both Mode I and Mode II on the right failure cross-section.

From the comparison between the failure cross-sections of Specimen W3 (Figure 48) and Specimen W4 (Figure 49), it follows that the internal hinge of Specimen W4 localized on the same cross-section as the internal hinge on Specimen W3 (9^th^ mortar bed joint from the left). In addition to this, a second internal hinge has formed for Specimen W4 on the 8^th^ mortar bed joint from the left (Figure 49b). This happened because the strapping provides an infinite degree of internal hyper-staticity to the isostatic static-scheme of the hinged supported I-beam, which allowed the formation of multiple plastic hinges on the cross-sections, without ever reaching a labile configuration (until the straps are broken).

**Figure 50:**
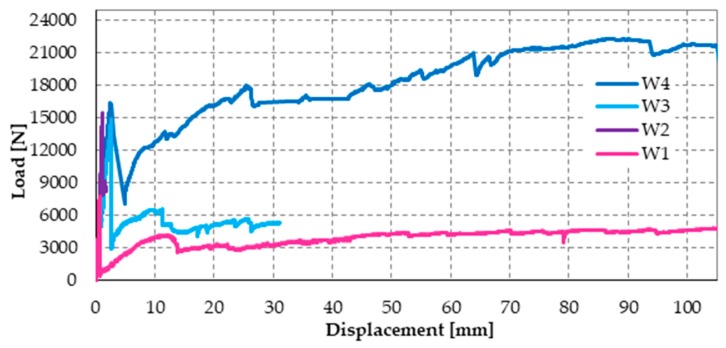
Comparison between the load/displacements diagrams of the four specimens.

The load/displacement diagram of Specimen W4 confirmed the general features of Specimen W1 and Specimen W3: the use of the steel straps made the retrofitted system extremely ductile, to such an extent that the specimen did not experienced collapse up to vertical displacements of the order of 10 cm (Figure 50). Even in the latter case, the operator had to stop the flexural tests to avoid instrumentation damages, while the specimen would have withstood further increases in displacement. Note that the load/displacement diagram of Specimen W3 in Figure 50 is much shorter than the diagrams of both Specimen W1 and Specimen W4 only because the instrumentation setup prompted the operator to interrupt the flexural test well in advance.

The comparison between the load/displacements diagrams of specimens W4 and W2 (Figure 50) suggests that the restoration of Specimen W3 was successful since the delamination load of Specimen W4 is comparable to the delamination load of Specimen W2 (retrofitted only with CFRP strips).

Moreover, Specimen W4 underwent delamination at 16.387 kN, while Specimen W3 underwent delamination at 14.904 kN. Therefore, the delamination load after restoration (Specimen W4) is 10% higher than the delamination load before restoration (Specimen W3). Since the number of transverse straps of Specimen W4 is higher than the number of transverse straps of Specimen W3, this confirms that a higher compression load on the CFRP strips delays delamination and increases the delamination load, as discussed in Section 3.2. In fact, a higher compression load increases the magnitude of the normal reaction force, **N**, in Figure 21c, which increases the distance between the resultant force acting on a CFRP strip, **Φ**, and the lateral surface of the cone of cohesive static friction.

Upon delamination of the stretched CFRP strip, the load withstood by Specimen W4 decreased abruptly, but to a lesser extent than for Specimen W3. In fact, the residual load withstood by Specimen W4 after delamination (7.083 kN) is about 236% of the residual load withstood by Specimen W3 (3.004 kN). Once again, the observed behavior depends on having increased the number of steel straps. In fact, in addition to the beneficial effect of the increased number of longitudinal straps (discussed above), the friction forces at the interface between CFRP strips and the masonry wall—activated by the compression loads provided by the transverse straps—counteract the sliding of the delaminated strip. Consequently, the transverse straps continue to tie the two flanges of the ideal I-beam together even after delamination. Moreover, the stiffness of the constraint established between the two flanges depends on the friction forces. Therefore, the greater the number of steel straps, the greater the friction forces and, consequently, the constraint stiffness. In conclusion, the greater the number of steel straps, the greater the residual load after delamination.

In the specific case of Specimen W4, the residual load after delamination is about 43% of the delamination load (57% load drop). Therefore, it is still too low to avoid instability or damage problems in the adjacent structural elements (of a real building) due to load redistribution. In fact, a load drop of 15% to 20% is the limit usually considered satisfactory to prevent such phenomena from occurring. Nevertheless, it is worth remembering that, in the spirit of a first investigation, the stiffness of the used ribbons is low (Figure 29), which is very far from the stiffness of the CAM ribbons (Figure 26). Therefore, the fact of having increased the residual load from 20% (Specimen W3) to 43% (Specimen W4) of the delamination load despite the low stiffness of the straps is a satisfactory result, since it certifies the effectiveness of the combined technique in any case. It is reasonable to think that the residual load will increase further if the combined technique uses high-strength steel ribbons, such as the ribbons of the CAM system, or steel wire ropes, as proposed in Section 5. In other words, the comparison between the results of Specimen W3 and W4 shows that it is possible to increase the residual load until the desired percentage of delamination load by increasing the stiffness and the number of straps. The materials and the number of straps most suitable in a real application are still under evaluation.

To complete the discussion on the load drop, it is necessary to analyze the post-delamination behavior of Specimen W4. In fact, the retrofitted system is extremely resilient (to such an extent as to be comparable to FRP wrapped columns [19]) and allows the regaining of the load after delamination. At the displacement value for which the operator stopped the flexural test (22.299 kN), the post-delamination load exceeded the delamination load by more than 36%. The reason for the load regaining lies in the positive contribution of increasing the number of steel straps, which becomes even more decisive in the post-delamination stage. After the initial decrease, the post-delamination load of Specimen W4 increases and maintains values that are much higher than those of Specimen W3. Even in the latter case, the increase in load depends on the I-beam behavior of the two CFRP strips, which was allowed after delamination by the friction forces developed at the interface with the masonry wall.

**Figure 51:**
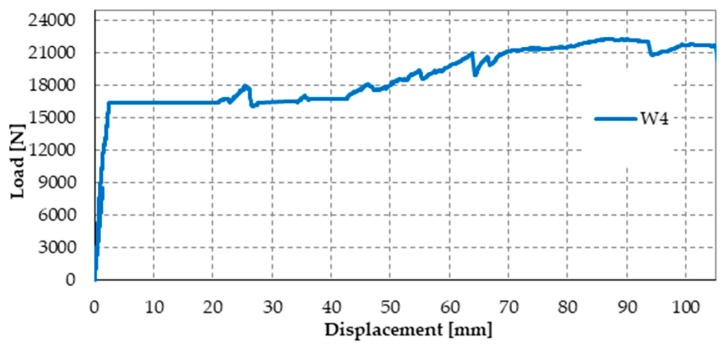
Load/displacement diagram of Specimen W4 in a flexural test performed in the load control.

From the structural point of view, the load regaining is very important, as it could affect the overall stability of a building. In the specific case of Specimen W4, there exists a vertical displacement value, for which the post-delamination load is equal to the delamination load and tends to increase further. Consequently, if the three-point bending flexural test takes place in the load control, the load/displacement diagram of Specimen W4 between the delamination load and the recovered load of post-delamination follows the horizontal path shown in Figure 51.

Since the retrofitted wall is able to find a new equilibrium configuration at a constant load and withstand further increases in the load, in a real application it would not trigger a load redistribution at delamination. Therefore, the adjacent structural elements would not experience overload due to the delamination of the CFRP strips.

Nevertheless, to find a new equilibrium configuration at constant load, the loaded cross-section undergoes an instantaneous increase in displacement, which could exceed the maximum displacement allowed by the ductility of the structure. In Figure 51, the increase in displacement, provided by the length of the horizontal path, is equal to 18 mm. However, this length decreases if the load drop also decreases by increasing the stiffness and the number of straps. Therefore, it is always possible to reduce the instantaneous displacement so that it is compatible with the ductility of the structure. This means that it is possible to design the retrofitting system so that the delamination of the CFRP strips does not coincide with the service limit.

Lastly, it is worth noting that the desired mechanical properties may differ for the longitudinal and transverse straps. In fact:

- The pre-tension of the transverse straps delays the delamination of the CFRP strips, by pushing the straps against the wall, as shown in Figure 36a,b, which blocks the CFRP strips. Since the (low) pre-tension is provided by imposing a relative displacement between the free ends of the straps, with the relative displacement set by the strapping tool, the pre-tension value and the consequent action on the CFRP strips depend on the stiffness of the straps (the greater the stiffness, the greater the pre-tension).
- The ductility of the longitudinal straps delays the failure of the retrofitted masonry wall, which allows the formation of multiple plastic hinges without ever reaching a labile configuration.

Therefore, having a high ductility is advantageous for the longitudinal straps, while it is disadvantageous for the transverse straps, at least as long as the strapping tool operates in the displacement control. Thus, it may be appropriate to use steel with different mechanical properties for the transverse and longitudinal straps.

Moreover, using more than one strap in the transverse direction is not as useful as in the longitudinal direction. Actually, strapping a steel ribbon on another strap reduces the pre-tension of the underlying strap. This is not important for the longitudinal straps, but is detrimental for the transverse straps. In fact, the advantage of using more than one longitudinal strap lies in the cross-sectional increase, which allows the specimen to withstand higher post-delamination loads, regardless of the pre-tension of straps. On the contrary, the transverse straps are all the more effective in pushing the CFRP straps against the wall as the greater the average pre-tension of the straps. With reference to Figure 50, this explains why the greater number of transverse straps of Specimen W4 increases the delamination load by only 10% when compared to Specimen W3, while the increase in the number of longitudinal straps is much more effective in modifying the post-delamination load.

## 5. A Further Combined Technique

The second combined technique proposed in this paper is an improvement of the technique discussed in Section 3.2. In fact, the second combined technique arises from the same ideal I-beam scheme that is at the base of the first combined technique, but differs from the latter for the materials used.

The reason that led the authors to change the materials of the tying system lies in the analysis of the results of Section 4. In particular, on the one hand, those first results showed that the CAM-like straps are actually able to provide an I-beam behavior that is particularly noticeable after delamination. However, on the other hand, the delamination load does not increase in a sensitive way. Assuming that the normal stresses at the interface between strips and straps increase the delamination load—due to the friction forces (Figure 20b)—it is, therefore, possible to conclude that stiffer strips would increase the delamination load. This suggested the authors to replace the steel ribbons with steel wire ropes.

**Figure 52:**
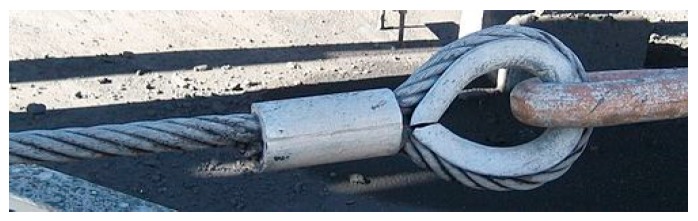
A wire rope terminated in a loop (Flemish eye) with a thimble and ferrule.

The use of steel wire ropes instead of steel ribbons leads the experimenters to face new problems to fasten the loose ends. The first problem concerns fraying since the end of a wire rope tends to fray readily, which do not allow easy connections. There are different ways of securing the ends of wire ropes to prevent fraying. The most common and useful type of end fitting for a wire rope is the Flemish eye, which consists in turning the end back to form a loop and fixing the loose end back on the wire rope (Figure 52).

If the wire rope terminates with a loop, there is a risk that it will bend too tightly when the loop is connected to a device that concentrates the load on a relatively small area. In these cases, a thimble installed inside the loop (Figure 52) is useful to preserve the natural shape of the loop and protect the cable from pinching and abrading on the inside of the loop. The thimble prevents the load from coming into direct contact with the wires.

**Figure 53:**
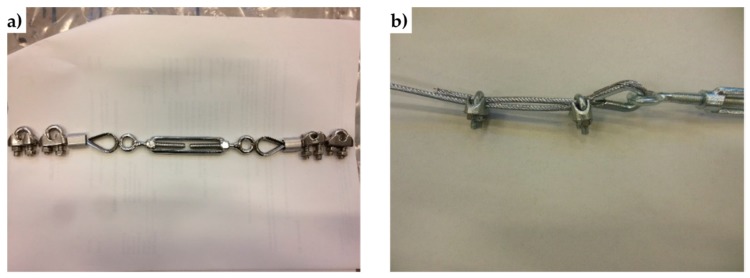
Devices used to fasten the loose ends of the steel wire ropes together, in the second combined technique: (**a**) overview of the sequence of devices, consisting of an eye-eye turnbuckle in the middle and a series of two clips, one ferrule, and one thimble on both sides. (**b**) How to install the clips, with the saddle portion of the clamp assembly placed against the “live” end.

In Figure 52, a ferrule fixes the loose end of the loop back to the wire rope. Another device for fixing the loose end of the Flemish eye is the wire rope clamp, which is also called a clip and consists of a U-shaped bolt, a forged saddle, and two nuts (Figure 53). The two layers of wire rope lie in the U-bolt. Then, the saddle fits above the ropes to the bolt (the saddle includes two holes to fit to the U-bolt) and the nuts secure the arrangement in place.

The function of the flat bearing seat and extended prongs of a clip (saddle) is to protect the live or stress-bearing end of the rope against crushing and abuse. Therefore, when installing clips, the saddle portion of the clamp assembly is placed against the load bearing or “live” end (Figure 53b), not against the non-load-bearing or “dead” side of the cable.

The number of clamps needed to terminate a wire rope, usually three or more, depends on the diameter of the wire rope. To choose the correct number of clamps for the second combined technique, the authors tested assemblies with three clamps (one ferrule and two clips, as in Figure 53a) and assemblies with two clamps (two clips, as in Figure 53b), finding the latter more performing for a 3 mm single strand zinc-coated wire rope.

The Flemish eyes of the second combined technique pass through the threaded eyebolts of a turnbuckle (Figure 53a). By rotating the metal frame of the eye-eye turnbuckle, it is possible to screw both eyebolts in or out simultaneously—without twisting the eyebolts or attached ropes—thus adjusting the tension of the loop-shaped ropes.

**Figure 54:**
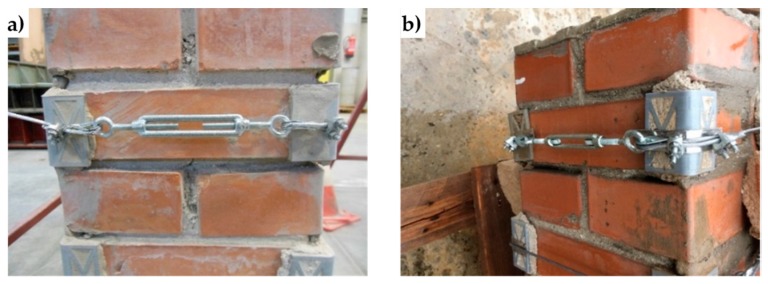
The clamping system of the second combined technique: (**a**) detail of the clamping system along the thickness of the brick wall (23 cm) and (**b**) detail of the two clips at the corner of the brick wall.

The clamping system of the second combined technique is longer than it is for the first combined technique. Nevertheless, it is possible to contain the second clamping system in the thickness of the wall specimens (Figure 54).

**Figure 55:**
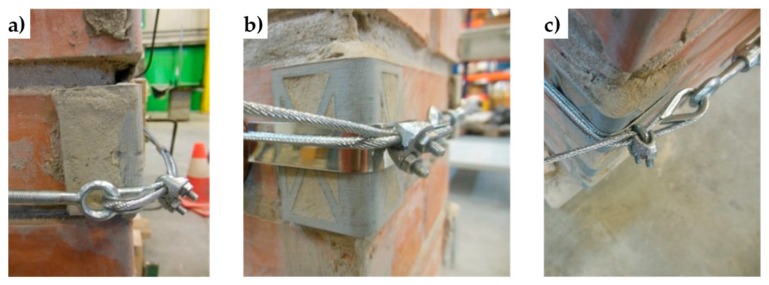
Rounded angles of the second combined technique: (**a**) indentation marks on a rounded angle after pre-tensioning and removal of the tying system, in the absence of a protective element between the steel wire rope and the PLA element. (**b**) Use of a steel ribbon to protect a PLA rounded angle (front view). (**c**) Use of a steel ribbon to protect a PLA rounded angle (top view).

**Figure 56:**
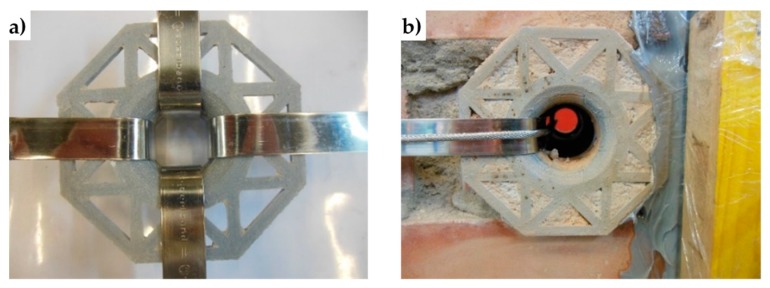
Funnel plates of the second combined technique: (**a**) arrangement of four pieces of steel ribbons to protect the rounded corners of the PLA funnel plates and (**b**) installation of a steel wire rope on a piece of steel ribbon, protecting the rounded corner of a PLA funnel plate.

To protect the rounded corners of the 3D printed elements, the second combined technique also uses some small pieces of steel ribbons, inserted between the steel wire ropes and the PLA elements (Figure 54b, Figure 55b,c, and Figure 56). In fact, in the absence of the pieces of steel ribbons, the pre-tensioning causes the steel wire ropes to indent the rounded corners of the 3D printed elements, as shown in Figure 55a.

The photos shown in Figure 53, Figure 54, Figure 55 and Figure 56 are part of a second experimental program on the combined techniques, performed at the LiSG laboratory of the University of Bologna, and still in progress. The preparation of specimens for the second experimental program did not entail particular technical difficulties. On the contrary, the possibility to unlock and reposition the straps after the first tightening made the strapping process more flexible.

**Figure 57:**
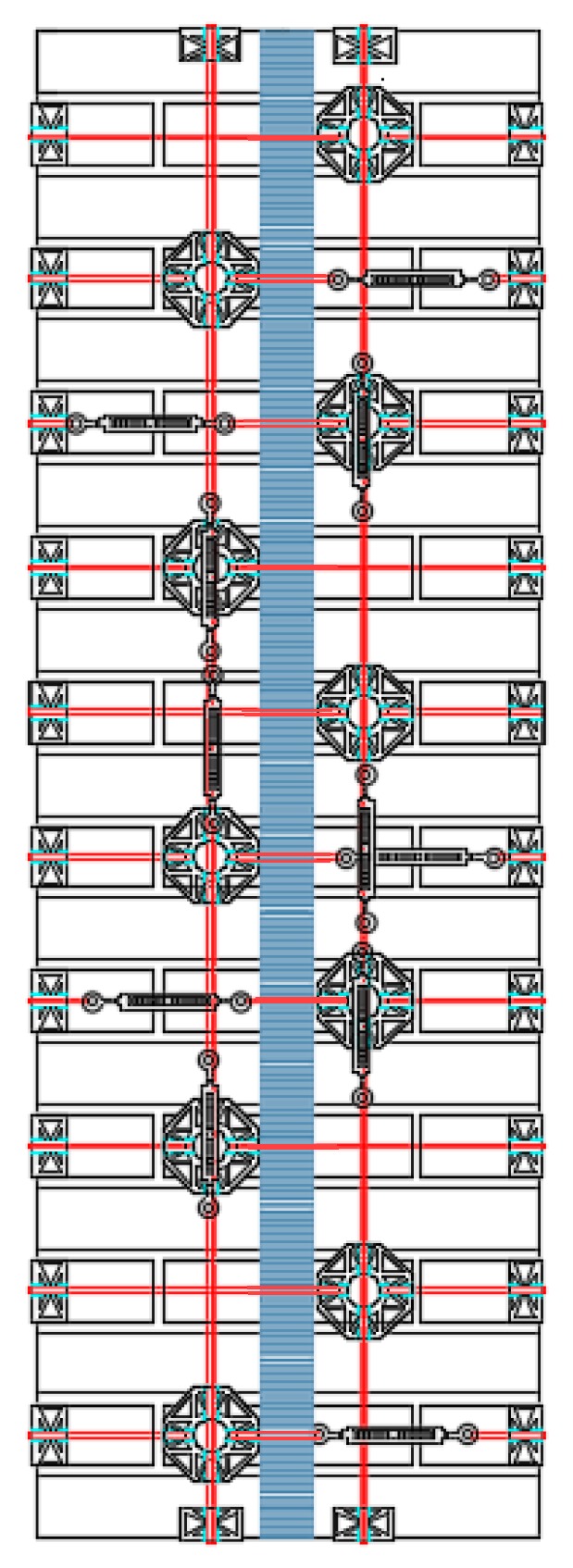
Scheme of the arrangement of the steel wire ropes in the second combined technique.

The arrangement of the steel wire ropes to tie the CFRP strips together by the second combined technique follows the general pattern shown in Figure 57, where the eye-eye turnbuckles clamp the steel wire ropes partly on the front side, partly on the rear side, and, partly, on the lateral sides. As with the first combined technique, the transverse ties pass over the CFRP strips and under the longitudinal ties.

## 6. Conclusions

In the present paper, the authors deepened the out-of-plane behavior of the retrofitting system introduced in Reference [17], which consist of a mechanical coupling between steel straps and CFRP strips. In particular, the authors have clarified how the transfer of stresses from the steel straps to the CFRP strips delays delamination on both sides of a bent beam, which allows a better exploitation of the strengthening properties of the CFRP strips.

Since the retrofitting system of the combined technique develops in the thickness of the masonry wall, it modifies the bending stiffness of the wall. In fact, due to the transverse link established by the mechanical coupling, two vertical CFRP strips on opposite sides of the wall behave like the two flanges of an ideal CFRP I-beam embedded into the wall. Since the bending stiffness of the ideal CFRP I-beam is much greater than the bending stiffness of the masonry wall, the effect of the ideal CFRP I-beam on the horizontal displacements of the wall is the same as that provided by an embedded buttress. In particular, the ideal CFRP I-beam is useful in terms of providing a bracing effect against the hammering action of floors, during an earthquake. Actually, compared to other bracing systems, the straps/strips technique is more appealing for more than one reason.

- It is an improvement of the CAM system, which is considered minimally invasive [7,8,9,10,11,12,13,14];
- The three-dimensional net of straps provides the building with a box-type behavior;
- Retrofitting does not excessively increase the total mass of the building, which limits the attraction of seismic forces;
- Once the masonry wall has cracked, the straps have exhausted their strengthening function but find a second use, starting to work as a device of safeguarding life.

Based on a theoretical analysis, the authors tested the flexural rigidity of masonry walls reinforced with the straps/strips technique by modifying the number of straps per loop to improve the strengthening system. The experimental results showed that the failure mechanism of the masonry walls with CFRP strips changed from brittle to ductile when the steel straps were applied. In particular, the two staggered nets of steel straps provided ductility to the crack propagation in Mode I, along the mortar bed joints. This allowed the formation of several plastic hinges without ever reaching the collapse of the specimens.

Another very interesting result of the experimentation concerns the resilience of the retrofitted system, which proved to be able to regain load after delamination of the CFRP strips, up to load values greater than the delamination load. Consequently, the failure load of the retrofitted system is greater than the delamination load. Furthermore, the retrofitted system continues to bear a load without causing a load redistribution, which is potentially dangerous for adjacent structural elements.

The analysis of the contribution of the longitudinal and transverse straps to the overall behavior has shown that it is possible to design the combined technique to meet the desired structural requirements. In particular, it is possible to decrease the load drop (flexural tests in a displacement control) and the instantaneous displacement (flexural tests in the load control) at delamination as desired.

Lastly, the authors noted that the longitudinal and transverse straps contribute in different ways to the mechanical behavior of the specimens. In fact, the transverse straps exploit the ribbon stiffness and the friction forces at the interface to increase the load, while the longitudinal straps take advantage of strength and ductility to improve the post-delamination behavior in terms of both load and ductility of the retrofitted wall.

Noting that the transverse straps are all more effective when their stiffness is larger suggests to the authors to replace the steel straps with steel wire ropes in order to improve the expected result of this experimental program and increase the out-of-plane strength of masonry walls.

The results of the straps/strips technique with steel wire ropes are currently under evaluation. What the authors can expect now is that the best choice to meet both needs of increasing the out-of-plane strength and safeguarding life is to use steel wire ropes for the transverse straps and steel ribbons for the longitudinal straps. In fact, highly pre-tensioned steel wire ropes will provide the wall with a bracing effect by counteracting the delamination of the CFRP strips and slightly pre-tensioned steel ribbons will act as dampers by allowing the formation of numerous dissipative plastic hinges, which avoid the structural collapse.

## 7. Future Developments

The next step of the research should be a cost-benefit analysis of the proposed solutions [20] to assess to which extent the presented solutions are sustainable. However, this analysis is premature now since the materials that optimize the performance of the combined technique in a real application are currently still under evaluation.

## Figures and Tables

**Table 1:** Geometric and mechanical characteristics of the six brick specimens.

Specimen	Dimensions [mm]	Weight [g]	Breaking Load [N]	Compressive Strength [N/mm^2^]	Normalized Compressive Strength [N/mm^2^]
PA1	55 × 54 × 55	296.1	116436	39.63165	34.47953
PA2	57 × 57 × 55	317.8	165730	50.91128	44.29281
PB1	55 × 53 × 55	297.5	146733	49.62439	43.17322
PB2	56 × 55 × 57	319.2	142681	46.09916	40.10627
PC1	56 × 53 × 56	310.5	144933	47.77687	41.56587
PC2	56 × 55 × 56	317.1	149422	48.14767	41.88848

**Table 2:** Geometric and mechanical characteristics of the mortar specimens.

Specimens of the Flexural Tests	Dimensions [mm]	Weight [g]	Breaking Load in Bending [N]	Flexural Strength [N/mm^2^]	Specimens of the Compression Tests	Breaking Load in Compression [N]	Compressive Strength [N/mm^2^]
P1	40 × 40 × 160	466.42	1758	4.12	P1A	30530	19.08
					P1B	36730	22.96
P2	40 × 40 × 160	469.81	1838	4.31	P2A	30980	19.36
					P2B	30930	19.33
P3	40 × 40 × 160	470.42	1443	3.38	P3A	27500	17.19
					P3B	28530	17.83
P4	40 × 40 × 160	459.63	1885	4.42	P4A	34544	21.59
					P4B	27730	17.33
P5	40 × 40 × 160	463.81	1990	4.66	P5A	33880	21.18
					P5B	35200	22.00
P6	40 × 40 × 160	462.01	1598	3.75	P6A	30400	19.00
					P6B	30450	19.03
